# Diversity-oriented synthesis of dihydrobenzoxazepinones by coupling the Ugi multicomponent reaction with a Mitsunobu cyclization

**DOI:** 10.3762/bjoc.10.16

**Published:** 2014-01-17

**Authors:** Lisa Moni, Luca Banfi, Andrea Basso, Alice Brambilla, Renata Riva

**Affiliations:** 1 Department of Chemistry and Industrial Chemistry, University of Genova, I-16146 Genova, Italy

**Keywords:** benzoxazepines, diversity-oriented synthesis, multicomponent reactions, Mitsunobu reaction, Ugi reaction

## Abstract

An operationally simple protocol for the synthesis of 2,3-dihydrobenzo[*f*][1,4]oxazepin-3-ones, based on an Ugi reaction of an *ortho*-(benzyloxy)benzylamine, glycolic acid, an isocyanide and an aldehyde, followed by an intramolecular Mitsunobu substitution was developed. The required *ortho*-(benzyloxy)benzylamines have been in situ generated from the corresponding azides, in turn prepared in high yields from salicylic derivatives.

## Introduction

Although the classical Ugi 4-component reaction (U-4CR) leads to acyclic peptide-like compounds, post-condensation cyclizations can afford a huge variety of drug-like heterocycles [1]. This is usually accomplished by introducing additional orthogonal functional groups in the starting components, taking advantage of the high tolerance of the U-4CR. In particular our group has been quite active in the last seven years in coupling the U-4CR with acyl or aliphatic substitution reactions [2], especially the intramolecular Mitsunobu reaction of alcohols with phenols or sulfonamides. By exploiting a single post-MCR transformation (the Mitsunobu reaction) it is possible to obtain several diverse heterocyclic scaffolds by installing the two additional groups in any of the four components and by varying the length of the spacers that connect them to the parent Ugi structure. In a previous paper [3] we have described an efficient access to dihydrobenzoxazinones by installing the alcohol moiety into the carboxylic acid and the nucleophile (a phenol) into the amine component. Our continuous interest in the use of isocyanide based MCRs in the synthesis of seven-membered heterocycles [4–7] has now prompted us to extend the methodology to the synthesis of 2,3-dihydrobenzo[*f*][1,4]oxazepin-3-ones **10**, which represent a typical drug-like scaffold, already demonstrated to be useful in medicinal chemistry [8–10].

## Results and Discussion

**Scheme 1:**
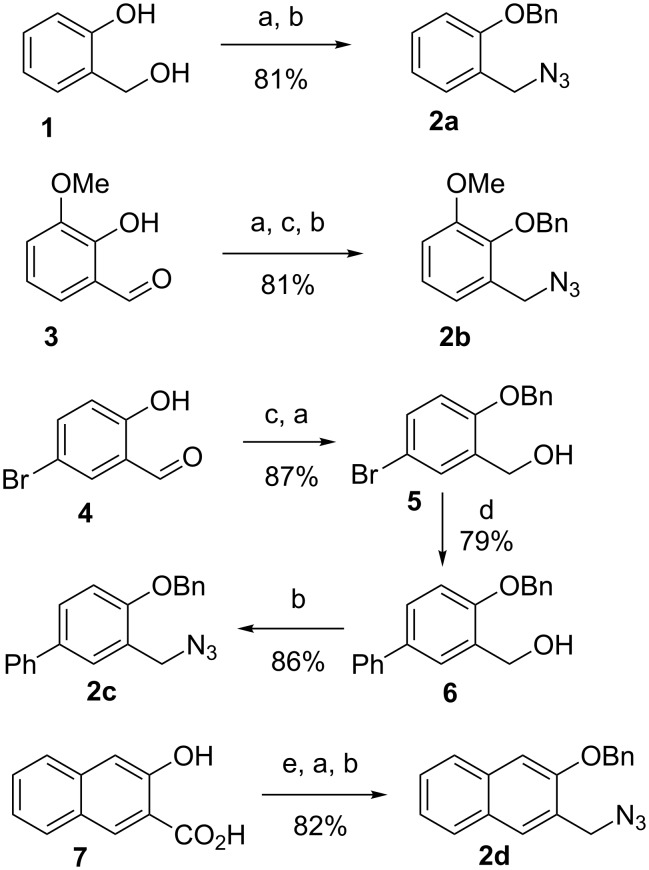
Synthesis of benzyl azides. a) BnBr, K_2_CO_3_, acetone or DMF, rt or 60 °C (for **2d**); b) 1) MsCl, Et_3_N, CH_2_Cl_2_, −10 °C; 2) NaN_3_, DMF, rt; c) NaBH_4_, MeOH, rt; d) PhB(OH)_2_, Cs_2_CO_3_, Pd(OAc)_2_, PPh_3_, 80 °C, 4.5 h; e) 1) MeOH, H_2_SO_4_, reflux; 2) LiAlH_4_, THF, rt.

Towards this goal we needed, as key components, *ortho*-hydroxybenzylamines. However, very few members of this family are commercially available, in contrast to the 2-hydroxyanilines employed in our previous synthesis of six-membered oxaza heterocycles [3]. On the other hand, various *ortho*-hydroxybenzyl alcohols are on the market or can be easily prepared from the corresponding salicylaldehydes or salicylic acids. Thus we decided to set up a general and efficient strategy to access the desired amines, through conversion of the benzyl alcohols into benzyl azides by nucleophilic substitution, followed by azide reduction. This strategy required in any case protection of the phenol moiety. As a matter of fact, in preliminary attempts, we found out that Ugi reactions employing free *para*-hydroxybenzylamines proceed in very poor yields (<25%), probably because of interference of the phenol moiety, which can act as internal nucleophile. For these reasons we decided to use the *O*-benzylated benzylamines as starting materials for the U-4CR, postponing the hydrogenolytic removal of the protecting group after the condensation. Four different benzyl azides **2a–d** were straightforwardly prepared in excellent yields from low cost starting materials **1**, **3**, **4**, and **7**, in all cases passing through the benzyl alcohols (Scheme 1). Apart from **2a** [11], they are all new compounds.

**Scheme 2:**
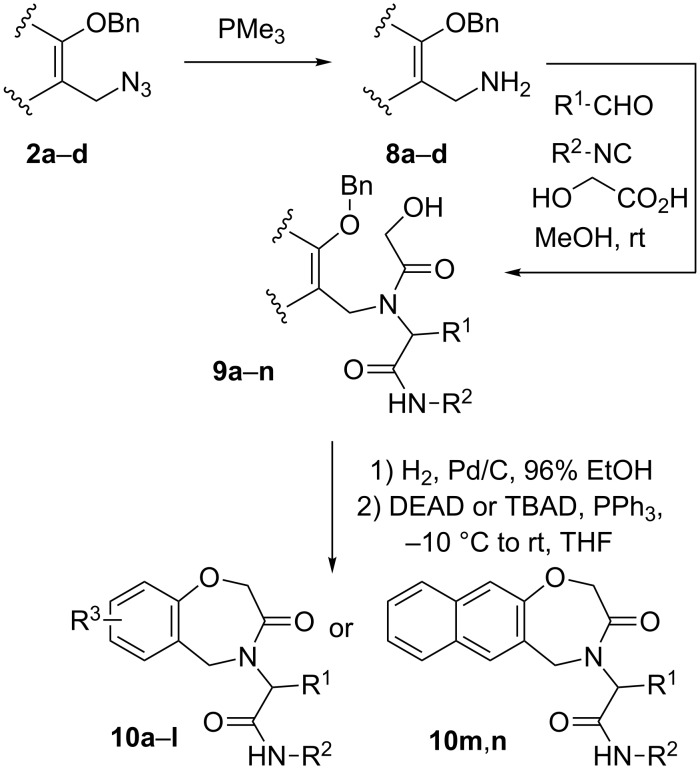
Synthesis of dihydrobenzoxazepinones **10**.

Initially we reduced azide **2a** with PPh_3_ and separated the amine from triphenylphosphine oxide by extracting it into acidic water. However, the amine recovery, after basification and a second extraction with an organic solvent, was never complete and the yields were poorly reproducible. This can be due to the sluggish and unpredictable hydrolysis of the intermediate phosphazene, and to the easy reaction of this electron-rich benzylamine with CO_2_ to give an insoluble carbamate. Skipping the extractive purification and directly using the crude amine in the ensuing sequence gave again erratic yields and was troublesome because of the difficult separation of the Ugi adducts **9** from triphenylphosphine oxide. We eventually found that the easiest and most efficient protocol involved reduction with Me_3_P, followed by evaporation of the solvent and by subsequent Ugi reaction on the crude. With Me_3_P, phosphazene hydrolysis was much faster and the phosphine oxide was much more easily separated by chromatography at the level of **9**. With these optimized conditions in hand, we performed a series of Ugi reactions using azides **2a–d**, glycolic acid, various aldehydes and isocyanides (Scheme 2). After a fast purification through a short silica gel column, the Ugi adducts **9a–n** were submitted to hydrogenolysis and, after catalyst removal and evaporation, to the final Mitsunobu cyclization. The overall sequence from starting benzyl azides **2a–d** to the heterocyclic products **10a–n** required just two evaporations, a filtration, and two chromatographic purifications.

Table 1 reports the results. Three diversity inputs have been varied and the overall yields are generally good (>45% overall yield from azides **2** to dihydrobenzoxazepinones **10**). In general diethyl azodicarboxylate (DEAD) or di-*t*-butyl azodicarboxylate gave similar results and the choice of the reagent was made only on the basis of the easiness of separation from the hydrazinocarboxylate side product. Only in the case of compound **10d** the overall yield was slightly lower. This was due to the concurrent formation of a 2-imidazoline through the 3-component Orru's reaction [12]. The imidazoline was even the major product when the reaction was carried out in methanol. Shifting to trifluoroethanol this side reaction was mostly, but not totally, suppressed.

**Table 1:** Scope of the synthesis of 2,3-dihydrobenzo[*f*][1,4]oxazepin-3-ones **10**.

Starting azide	R^1^	R^2^	R^3^	Ugi time	Yield (**9**)^a^	Mitsunobu reagent^b^	Yield (**10**)^c^	Final product	Overall yield

**2a**	*c-*Hex	*t-*Bu	H	22 h	92%	DEAD	82%	**10a**	75%
**2a**	Ph	*c-*Hex	H	24 h	82%	DEAD	70%	**10b**	57%
**2a**	3,4-(OCH_2_O)C_6_H_4_	*t-*Bu	H	24 h	92%	DEAD	80%	**10c**	74%
**2a**	3,4,5-(OMe)C_6_H_3_	EtO_2_C-CH_2_	H	40 h	46%^d^	DEAD	59%	**10d**	27%
**2a**	Ph	*n-*Bu	H	48 h	63%	DEAD	85%	**10e**	54%
**2a**	iBu	*c-*Hex	H	67 h	86%	DEAD	80%	**10f**	69%
**2b**	Et	Me	6-OMe	19 h	54%	DEAD	98%	**10g**	53%
**2b**	*t-*Bu	Bn	6-OMe	120 h	74%	TBAD	92%	**10h**	68%
**2b**	Ph	*t-*Bu	6-OMe	42 h	84%	TBAD	93%	**10i**	78%
**2b**	Ph	2,6-MeC_6_H_4_	6-OMe	48 h	89%	TBAD	66%	**10j**	59%
**2c**	*c-*Hex	*t-*Bu	4-Ph	144 h	77%	DEAD	93%	**10k**	72%
**2c**	iBu	2,6-MeC_6_H_4_	4-Ph	96 h	89%	DEAD	54%	**10l**	48%
**2d**	3,4,5-(OMe)C_6_H_3_	*n-*Bu		72 h	66%	TBAD	92%	**10m**	61%
**2d**	*t-*Bu	Bn		72 h	68%	TBAD	65%	**10n**	45%

^a^Isolated yields (after chromatography) from azides **2a–d**. ^b^DEAD = diethyl azodicarboxylate; TBAD = di-*tert*-butyl azodicarboxylate. ^c^Isolated yields (after chromatography) from Ugi adducts **9a–n**. ^d^In this case trifluoroethanol was used as solvent.

## Conclusion

In conclusion, we have reported a further example of a synthesis of seven-membered heterocycles by coupling the Ugi multicomponent reaction with an intramolecular Mitsunobu reaction. This operationally simple protocol opens a straightforward route to 2,3-dihydrobenzo[*f*][1,4]oxazepin-3-ones **10** starting from 2-(benzyloxy)benzyl azides, in turn accessible from variously substituted salicylic aldehydes or acids. We were able to use directly the azides as input in the Ugi reaction without the need to isolate the intermediate amine. Since complex amines are often synthesized from the corresponding alcohols via substitution with an azide anion, this one-pot procedure can be useful in further expanding the scope of the Ugi reaction, in addition to the recently reported in situ generation of aldehydes/imines [13–15] and isocyanides [16–17].

## Experimental

**Typical procedure for the synthesis of dihydrobenzoxazepinones 10a–n.** A solution of benzyl azide **2** (1 mmol) in dry THF (3 mL) was treated with trimethylphosphine (1 M solution in toluene, 1.1 mmol) and stirred for 2 h at room temperature. Then water (4 mmol, 68 μL) was added and the mixture was further stirred for 1 h. The solvent was evaporated and the crude taken up in dry methanol (2.5 mL) and treated with 3 Å powdered molecular sieves (50 mg). Glycolic acid (1.2 mmol) and the appropriate aldehyde and isocyanide (1.2 mmol each) were added, and the solution was stirred at room temperature for the time reported in Table 1. After evaporation, the residue was chromatographed with 220–400 mesh silica gel and PE/EtOAc (the ratio depends on the polarity of **9**). The fractions containing the Ugi product **9** were evaporated to dryness and weighted. afterwards **9** was taken up in 96% ethanol (5 mL/mmol), treated with 10% Pd/C (130 mg/mmol) and hydrogenated at 1 atm and room temperature for 20–40 h (until the reaction was complete (tlc control)). After filtration of the catalyst and evaporation of the solvent, the crude was taken up in dry THF (5 mL/mmol), cooled to −10 °C, and treated with PPh_3_ (1.5 equiv) and DEAD or TBAD (1.5 equiv). After stirring for 1 h, the temperature was allowed to rise to rt, and the solution stirred overnight. After evaporation the crude was chromatographed with 220–400 mesh silica gel and PE/EtOAc (the ratio depends on the polarity of **10**) to give the pure final product.

## Supporting Information

File 1 Experimental procedure, characterization data and copies of ^1^H and ^13^C spectra of all new compounds.
