# Supplementary material for: Diversity-oriented synthesis of dihydrobenzoxazepinones by coupling the Ugi multicomponent reaction with a Mitsunobu cyclization
Source: Beilstein J Org Chem. 2014 Jan 17;10:209–12. doi: 10.3762/bjoc.10.16 (PMC3943941; doi:10.3762/bjoc.10.16)
Supplement: File 1 — Experimental procedure, characterization data and copies of 1H and 13C spectra of all new compounds. [file Beilstein_J_Org_Chem-10-209-s001.pdf]

Supporting Information

for

Diversity-oriented synthesis of dihydrobenzoxazepinones by coupling the Ugi multicomponent reaction with a Mitsunobu cyclization

Lisa Moni, Luca Banfi, Andrea Basso, Alice Brambilla and Renata Riva*

Address: Department of Chemistry and Industrial Chemistry, University of Genova, I-16146 Genova, Italy

E-mail: Renata Riva* - riva@chimica.unige.it

*Corresponding author

Experimental procedure, characterization data and copies of ^{1}H and ^{13}C spectra of all new compounds

Table of Contents

1. Experimental details and characterization data of new compounds S2
2. NMR Spectra of new compounds S14

1) Experimental details and characterization of new compounds

General remarks

NMR spectra were taken at r.t. in $CDCl_3$ at 300 MHz (^{1}H), and 75 MHz (^{13}C), using, as internal standard, TMS (^{1}H NMR: 0.000 ppm) or the central peak of $CDCl_3$ (^{13}C: 77.02 ppm). Chemical shifts are reported in ppm (δ scale). Coupling constants are reported in Hertz. Peak assignments were made with the aid of gCOSY and gHSQC experiments. In ABX system, the proton A is considered upfield and B downfield. GC-MS were carried out using an HP-1 column (12 m long, 0.2 mm wide), electron impact at 70 eV, and a mass temperature of about 170°C. Only m/z > 33 were detected. All analyses were performed (unless otherwise stated) with a constant He flow of 0.9 ml/min with initial temp. of 100°C, init. time 2 min, rate 20°C/min, final temp. 280°C, inj. temp. 250°C, det. temp. 280°C. IR spectra were recorded as $CHCl_3$ solutions or directly on solid, oil or foamy samples, with the ATR (attenuated total reflectance) technique. TLC analyses were carried out on silica gel plates and viewed at UV (254 nm) and developed with Hanessian stain (dipping into a solution of $(NH_4)_4MoO_4\cdot 4\,H_2O$ (21 g) and $Ce(SO_4)_2\cdot 4\,H_2O$ (1 g) in H_2SO_4 (31 ml) and H_2O (469 ml) and warming) or, only to detect free amines after Staudinger reduction, with ninhydrin (900 mg of ninhydrin in 300 ml *n*BuOH and 9 ml AcOH, followed by warming). R_f were measured after an elution of 7-9 cm. Column chromatographies were done with the "flash" methodology using 220-400 mesh silica. Petroleum ether (40-60°C) is abbreviated as PE. In extractive work-up, aqueous solutions were always reextracted thrice with the appropriate organic solvent. Organic extracts were always dried over Na_2SO_4 and filtered, before evaporation of the solvent under reduced pressure. All reactions using dry solvents were carried out under a nitrogen (or argon if specified) atmosphere. Petroleum ether (40-60°C) is abbreviated as PE. In extractive work-up, aqueous solutions were always re-extracted thrice with the appropriate organic solvent. Organic extracts were always dried over Na_2SO_4 and filtered, before evaporation of the solvent under reduced pressure. All reactions using dry solvents were carried out under a nitrogen atmosphere, unless otherwise stated.

(2-(Benzyloxy)phenyl)methanol

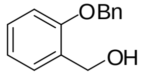

It was prepared in 94% yield from commercially available 2-(hydroxymethyl)phenol, following the reported procedure [1]. The physical and spectral data were in agreement with those reported [1-2].

1-(Azidomethyl)-2-(benzyloxy)benzene (2a)

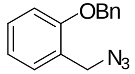

2a

A solution of 2-((benzyloxy)phenyl)methanol (2.00 g, 9.34 mmol) in dry dichloromethane (20 mL) was cooled to –30°C and treated with Et_3N (1.70 mL, 12.15 mmol) and methanesulphonyl chloride (868 μL, 11.21 mmol). The mixture was stirred at –30°C for 5 h and at r.t. for 30 min (due to

hydrolitic instability of the mesylate it is not possible to follow the reaction by TLC). Then the solvent was evaporated. The crude residue was taken up in dry DMF (20 mL) and treated with sodium azide (1.286 g, 19.62 mmol). The mixture was stirred at r.t. for 20 h. Then it was diluted with water (50 mL) and extracted three times with Et_2O. The organic phases were washed with water and brine, evaporated and chromatographed (PE/Et_2O 98:2 to 96:4) to give pure **2a** as a colourless oil (1.92 g, 86%). The physical and spectral data were in agreement with those reported [3].

1-(Azidomethyl)-2-(benzyloxy)-3-methoxybenzene (2b)

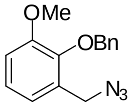

2b

It was prepared from known (2-(Benzyloxy)3-methoxyphenyl)methanol (in turn prepared in quantitative yield by reduction of the corresponding aldehyde [4]) following the same procedure employed for **2a**. White foam. Yield 81%. R_f 0.49 (PE/AcOEt 9:1). ^{1}H NMR (300 MHz, $CDCl_3$, 25 °C) δ 3.90 (3 H, s, C*H*$_3$); 4.26 (2 H, s, C*H*$_2$N$_3$); 5.07 (2 H, s, C*H*$_2$Ph), 6.90 (1 H, dd, *H*-4 or *H*-6, J 1.5, 7.8); 6.95 (1 H, dd, *H*-4 or *H*-6, J 1.5, 8.1); 7.08 (1 H, t, *H*-5, J 7.8); 7.29-7.48 (5 H, m, benzyl C*H*). ^{13}C NMR (75 MHz, $CDCl_3$, 25 °C) δ 49.8 (*C*H$_2$N$_3$); 55.9 (O*C*H$_3$); 75.1 (*C*H$_2$Ph); 112.8, 121.6, 124.3, 128.1, 128.3 (x2), 128.4 (aromatic *C*H); 129.7, 137.5, 146.0, 152.8 (aromatic quat.). I.r. (ATR): ν_{max} 2091, 1587, 1497, 1479, 1454, 1439, 1376, 1342, 1275, 1214, 1180, 1077, 978, 915, 884, 860, 784, 747, 696, 612 cm^{-1}. HRMS (ESI+) m/z: $[M + H]^+$ Calcd for $C_{15}H_{16}N_3O_2$ 270.1243; Found 270.1240.

(2-(Benzyloxy)-5-phenyl)phenyl)methanol (6)

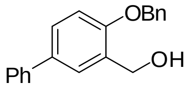

6

A solution of (2-(benzyloxy)-5-bromophenyl)methanol [5] (400 mg, 1.36 mmol), phenylboronic acid (249.4 mg, 2.05 mmol) and PPh_3 (53.6 mg, 205 μmol) in dry THF (10 ml) is placed in a pressure tube. A solution of Cs_2CO_3 (889 mg, 2.73 mmol) in H_2O (2 mL) is added and the sistem is flushed with argon. After addition of the catalyst ($Pd(OAc)_2$) (15 mg, 78 μmol) the tube is sealed and heated at 90°C in a pre-heated oil bath. After 4 h the system is cooled and the mixture poured into saturated aqueous NH_4Cl (140 mL) and extracted three times with CH_2Cl_2. Evaporation followed by chromatography (PE/Et_2O 66:34) gave pure **6** as a white solid (313 mg, 79%). M.p.: 100.5-101.2 °C. R_f 0.27 (PE/Et_2O 66:34). ^{1}H NMR (300 MHz, $CDCl_3$, 25 °C) δ 2.34 (1 H, t, O*H*, J 5.4); 4.80 (2 H, d, C*H*$_2$OH, J 5.4); 5.16 (2 H, s, C*H*$_2$Ph); 7.02 (1 H, d, *H*-3, J 8.1); 7.26-7.46 (8 H, m); 7.48 (1 H, dd, *H*-4, J 2.1, 8.1); 7.53-7.58 (3 H, m). ^{13}C NMR (75 MHz, $CDCl_3$, 25 °C) δ 62.2 (*C*H$_2$OH); 70.2 (*C*H$_2$Ph); 112.0 (*C*-3); 126.8 (x3), 127.3 (x2), 127.4, 127.6, 128.2, 128.7 (x4) (other aromatic *C*H); 129.8, 134.1, 136.7, 140.6, 156.1 (aromatic quat.). I.r. (ATR): ν_{max} 3273, 3063, 3035, 2859, 1608, 1514, 1488, 1452, 1381, 1360, 1309, 1289, 1274, 1249, 1177, 1128, 1079, 1047, 1035, 1024, 1008, 920, 905, 891, 841, 803, 772, 759, 731, 691, 632, 618 cm^{-1}. GC-MS: R_t 13.27 min; *m/z* 290 (M^+, 0.04%) 182 (28.5) 153 (5.0) 152 (5.5) 92 (8.3) 91 (100) 65 (10.1). HRMS (ESI+) m/z: $[M + H]^+$ Calcd for $C_{20}H_{19}O_2$ 291.1385; Found 291.1381.

1-(Azidomethyl)-2-(benzyloxy)-5-phenylbenzene (2c)

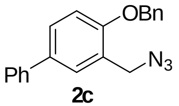

2c

It was prepared from **6** in 86% yield following the same procedure employed for **2a**. White solid. M.p.: 73.5-74.5 °C. R_f 0.36 (PE/Et_2O 97:3). ^1H NMR (300 MHz, $CDCl_3$, 25 °C) δ 4.48 (2 H, s, CH_2N_3); 5.17 (2 H, s, CH_2Ph), 7.04 (1 H, d, *H*-3, J 9.3); 7.27-7.60 (12 H, m, other aromatic C*H*). ^{13}C NMR (75 MHz, $CDCl_3$, 25 °C) δ 50.2 (*C*H_2N_3); 70.3 (*C*H_2Ph); 112.2 (*C*-2), 124.6 (*C*-1), 126.8 (x2), 126.9, 127.3 (x2), 128.1, 128.2, 128.7 (x2), 128.78 (x2), 128.84 (other aromatic *C*H); 134.1, 136.7, 140.3, 156.1 (other aromatic quat.). I.r. (ATR): ν_{max} 2116, 1610, 1586, 1508, 1484, 1462, 1456, 1450, 1383, 1350, 1295, 1279, 1271, 1250, 1242, 1228, 1179, 1134, 1077, 1054, 1015, 999, 951, 908, 899, 856, 843, 827, 767, 758, 740, 714, 696, 625, 605 cm^{-1}. GC-MS: R_t 13.39 min; *m*/*z* 315 (M^+, 0.002%) 196 (7.9) 182 (8.1) 115 (5.1) 92 (9.3) 91 (100) 65 (12.7).HRMS (ESI+) m/z: $[M + H]^+$ Calcd for $C_{20}H_{18}N_3O$ 316.1450; Found 316.1451.

2-(Azidomethyl)-3-(benzyloxy)naphthalene (2d)

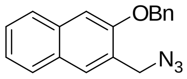

2d

It was prepared in 98% yield from known (3-(benzyloxy)naphthalen-2-yl)methanol [6-7] following the same procedure employed for **2a**. In this case no chromatography was needed. White solid. M.p.: 68.8-72.2 °C. R_f 0.82 (PE/Et_2O 75:25). ^1H NMR (300 MHz, $CDCl_3$, 25 °C) δ 4.57 (2 H, s, CH_2N_3); 5.24 (2 H, s, CH_2Ph), 7.24 (1 H, s, *H*-4); 7.32-7.47 (5 H, m, benzyl C*H*); 7.47-7.53 (2 H, m, *H*-6 and *H*-7); 7.72 (1 H, d, *H*-5 or *H*-8, J 8.1); 7.77 (1 H, s, *H*-1); 7.79 (1 H, d, *H*-5 or *H*-8, J 8.1). ^{13}C NMR (75 MHz, $CDCl_3$, 25 °C) δ 50.6 (*C*H_2N_3); 70.2 (*C*H_2Ph); 106.7 (*C*-4); 124.2, 126.6, 126.7, 127.4 (x2), 127.7, 128.1, 128.7 (x2), 129.3 (other aromatic *C*H); 125.8, 128.6, 134.4, 136.6, 154.7 (other aromatic quat.). I.r. (ATR): ν_{max} 2095, 2081, 1634, 1602, 1506, 1466, 1455, 1435, 1399, 1380, 1345, 1282, 1243, 1181, 1159, 1105, 1008, 995, 925, 889, 863, 831, 762, 751, 722, 703, 689, 621 cm^{-1}. GC-MS: R_t 12.55 min; *m*/*z* 289 (M^+, 9.3%) 261 (9.8) 260 (6.7) 170 (10.7) 156 (13.9) 128 (14) (7.9) 115 (7.0) 92 (7.9) 91 (100) 65 (11.4) 44 (11.3) 40 (9.9). HRMS (ESI+) m/z: $[M + H]^+$ Calcd for $C_{18}H_{16}N_3O$ 290.1293; Found 290.1296.

N-(*tert*-Butyl)-2-cyclohexyl-2-(3-oxo-2,3-dihydrobenzo[*f*][1,4]oxazepin-4(5*H*)-yl)acetamide (10a)

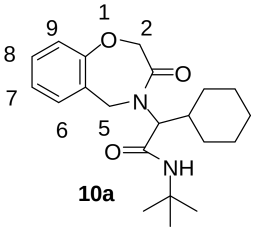

10a

White solid. M.p.: 135.4-136.8 °C. R_f 0.32 (PE/AcOEt 80:20). ^{1}H NMR (300 MHz, $CDCl_3$, 25 °C) δ 0.79-1.02 (2 H, m); 1.07-1.52 (3 H, m); 1.21 (9 H, s, (C(CH_3)$_3$)); 1.57-1.83 (5 H, m); 2.06 (1 H, tq, C*H*-CH-C=O, J_t 3.2, J_q 11.0); 4.57 (1 H, d, C*H*-C=O, J 11.1); 4.64 and 4.72 (2 H, AB syst., *H*-5, J_{AB} 15.3); 4.71 and 4.81 (2 H, AB syst., *H*-2, J_{AB} 15.3); 5.74 (1 H, s, N*H*); 6.96-7.04 (2 H, m, *H*-9, *H*-7); 7.17-7.28 (2 H, m, *H*-7, *H*-8). ^{13}C NMR (75 MHz, $CDCl_3$, 25 °C) δ 25.4, 25.6, 26.2, 28.8, 29.6 (*C*H_2 of cyclohexyl); 28.4 (C(*C*H_3)$_3$); 35.6 (*C*H-CH-C=O); 43.9 (*C*-5); 51.3 (*C*(CH_3)$_3$); 61.9 (*C*H-C=O); 71.6 (*C*-2); 119.4 (*C*-9); 123.3 (*C*-7); 127.3 (*C*-5a); 129.5, 129.7 (*C*-8 and *C*-6); 157.2 (*C*-9a); 168.6, 169.6 (*C*=O). I.r. ($CHCl_3$): ν_{max} 3419, 2928, 2851, 1670, 1618, 1450, 1364, 1327, 1230, 1110, 1046 cm^{-1}. GC-MS: R_t 12.58 min; *m*/*z* 358 (M^+, 3.6%) 286 (7.8) 285 (31.8) 258 (84.1) 257 (10.0) 203 (14.3) 198 (14.1) 197 (100) 176 (20.6) 164 (31.6) 163 (9.8) 162 (28.8) 154 (5.5) 148 (19.2) 147 (5.0) 141 (12.9) 134 (10.9) 123 (5.4) 122 (7.1) 121 (7.7) 120 (6.5) 119 (13.3) 118 (5.8) 115 (16.7) 112 (11.8) 110 (6.1) 107 (37.2) 106 (9.0) 98 (17.5) 95 (92.2) 93 (11.6) 91 (37.4) 89 (9.4) 85 (7.1) 81 (8.9) 80 (5.9) 78 (21.0) 77 (19.9) 67 (30.7) 65 (8.5) 57 (48.2) 55 (33.6) 42 (72.6) 41 (53.9). HRMS (ESI+) m/z: $[M + H]^+$ Calcd for $C_{21}H_{31}N_2O_3$ 359.2335; Found 359.2336.

***N*-Cyclohexyl-2-(3-oxo-2,3-dihydrobenzo[*f*][1,4]oxazepin-4(5*H*)-yl)-2-phenylacetamide (10b)**

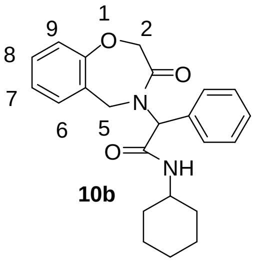

10b

White foam. R_f 0.64 (PE/AcOEt 50:50). ^{1}H NMR (300 MHz, $CDCl_3$, 25 °C) δ 1.00-1.20 (3 H, m); 1.20-1.45 (2 H, m); 1.52-1.75 (3 H, m); 1.78-2.02 (2 H, m); 3.84 (1 H, dtt, C*H*NH, J_t 3.7, 10.8, J_d 7.8); 4.25 (1 H, d, *H*-5, J 15.3); 4.68 and 4.87 (2 H, AB syst., *H*-2, J_{AB} 15.6); 4.78 (1 H, d, *H*-5, J 15.3); 5.90 (1 H, d, N*H*, J 7.8); 6.38 (1 H, s, C*H*-C=O); 6.63 (1 H, dd, *H*-6, J 7.5, 1.2); 6.87 (1 H, dt, *H*-7, J_t 7.4, J_d 0.9); 6.98 (1 H, d, *H*-9, J 7.5); 7.20 (1 H, dt, *H*-8, J_t 8.1, J_d 1.2); 7.33 (5 H, s). ^{13}C NMR (75 MHz, $CDCl_3$, 25 °C) δ 24.7 (x2), 25.4, 32.7, 32.9 (cyclohexyl *C*H); 45.8 (*C*-5); 48.6 (*C*HNH); 60.5 (*C*H-C=O); 72.0 (*C*-2); 119.5 (*C*-9); 123.5 (*C*-7); 128.3 (*C*-5a); 128.5 (*C* para of Ph); 128.8, 129.0 (*C* ortho and meta of Ph); 129.1 (*C*-6); 129.6 (*C*-8); 134.9 (quat. of Ph); 157.4 (*C*-9a); 168.0, 169.8 (*C*=O). I.r. ($CHCl_3$): ν_{max} 3413, 3330, 3001, 2925, 2852, 2702, 1660, 1488, 1450, 1321, 1219, 1022, 919, 661 cm^{-1}. GC-MS: R_t 12.203 min; *m*/*z* 378 (M^+, 0.2%) 253 (11.4) 252 (24.3) 218 (16.9) 217 (100.0) 162 (13.2) 146 (10.7)

136 (14.1) 121 (5.8) 119 (11.3) 118 (50.3) 107 (8.3) 106 (9.4) 104 (8.5) 92 (9.6) 91 (55.9) 90 (7.4) 89 (7.0) 83 (5.8) 78 (7.0) 77 (10.2) 65 (4.7) 55 (12.8) 41 (9.5). HRMS (ESI+) m/z: $[M + H]^+$ Calcd for $C_{23}H_{27}N_2O_3$ 379.2022; Found 379.2016.

2-(Benzo[*d*][1,3]dioxol-5-yl)-*N*-(*tert*-butyl)-2-(3-oxo-2,3-dihydrobenzo[*f*][1,4]oxazepin-4(5*H*)-yl)acetamide (10c)

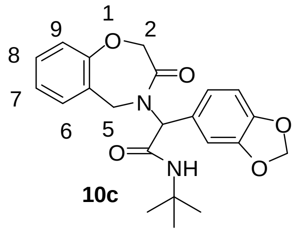

10c

White solid. M.p.: 194.9-195.9 °C. R_f 0.65 (PE/AcOEt 50:50). ^{1}H NMR (300 MHz, $CDCl_3$, 25 °C) δ 1.34 (9 H, s, C(CH_3)$_3$); 4.26 (1 H, d, *H*-5, J 15.6); 4.67 and 4.91 (2 H, AB syst., *H*-2, J_{AB} 15.5); 4.81 (1 H, d, *H*-5, J 15.6); 5.61 (1 H, s, N*H*); 5.97 and 5.98 (2 H, AB syst. OCH_2O, J_{AB} 1.4); 6.17 (1 H, s, C*H*-C=O); 6.68 (1 H, dd, *H*-6, J 7.5, 1.5); 6.76-6.85 (3 H, m, C*H* of benzodioxole); 6.90 (1 H, dt, *H*-7, J_t 7.5, J_d 1.2); 6.98 (1 H, dd, *H*-9, J 8.1, 1.2); 7.20 (1 H, dt, *H*-8, J_t 7.8, J_d 1.2). ^{13}C NMR (75 MHz, $CDCl_3$, 25 °C) δ 28.6 (C(*C*H_3)$_3$); 45.7 (*C*-5); 51.9 (*C*NH); 60.7 (*C*H-C=O); 71.9 (*C*-2); 101.3 (O-*C*-O); 108.4, 109.6, 119.6 (*C*H benzodioxole); 122.8 (*C*-9); 123.3 (*C*-7); 127.9, 128.6 (*C*-5a and quat. benzodioxole); 129.1 (*C*-6); 129.7 (*C*-8); 147.7, 148.0 (quat. of benzodioxole); 157.4 (*C*-9a); 168.3, 169.7 (*C*=O). I.r. (ATR): ν_{max} 3299, 2968, 1680, 1640, 1608, 1548, 1489, 1467, 1456, 1440, 1353, 1256, 1217, 1199, 1170, 1114, 1104, 1065, 1035, 1021, 938, 926, 862, 808, 797, 747, 727, 705, 650, 623 cm^{-1}. GC-MS: R_t 14.94 min; *m*/*z* 396 (M^+, 7.2%) 323 (25.7) 297 (39.4) 296 (59.3) 235 (49.8) 217 (40.2) 190 (15.0) 179 (10.5) 176 (40.8) 175 (5.4) 163 (13.9) 162 (100) 161 (13.3) 150 (14.2) 149 (20.3) 148 (37.1) 147 (17.7) 146 (32.3) 136 (46.2) 135 (84.7) 134 (11.2) 133 (6.2) 132 (17.3) 121 (9.8) 120 (7.8) 119 (8.9) 118 (5.6) 107 (14.1) 105 (9.8) 104 (22.1) 93 (5.3) 92 (5.9) 91 (27.1) 90 (7.3) 89 (9.6) 79 (5.7) 78 (12.6) 77 (26.9) 76 (12.5) 65 (14.2) 63 (7.6) 57 (28.3) 51 (9.7) 44 (6.7) 42 (6.4) 41 (18.7) 40 (7.6) 39 (8.5). HRMS (ESI+) m/z: $[M + H]^+$ Calcd for $C_{22}H_{25}N_2O_5$ 397.1763; Found 397.1758.

Ethyl 2-(2-(3-oxo-2,3-dihydrobenzo[*f*][1,4]oxazepin-4(5*H*)-yl)-2-(3,4,5-trimethoxyphenyl)acetamido)acetate (10d)

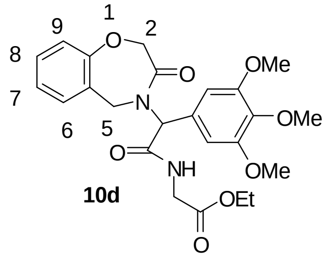

10d

White solid. M.p.: 143.6-149.7 °C. R_f 0.38 (PE/AcOEt 34:66). ^{1}H NMR (300 MHz, $CDCl_3$, 25 °C) δ 1.29 (3 H, t, CH_3CH_2, J 7.0); 3.70 (6 H, s, OCH_3); 3.84 (3 H, s, OCH_3); 3.93 (1 H, dd, C*H*HCO_2Et, J 5.1, 18.0); 4.07-4.30 (3 H, m, CH_2CH_3 and C*H*HCO_2Et); 4.18 (1 H, d, *H*-5, J 15.6); 4.64

and 5.02 (2 H, AB syst., *H*-2, J_{AB} 15.0); 4.94 (1 H, d, *H*-5, J 15.6); 6.37 (1 H, s, C*H*-C=O); 6.41 (1 H, broad s, N*H*); 6.57 (2 H, s, C*H* trimethoxyphenyl); 6.64 (1 H, dd, *H*-6, J 7.5, 1.2); 6.87 (1 H, dt, *H*-7, J_t 7.4, J_d 1.2); 6.98 (1 H, dd, *H*-9, J 8.1, 10.9); 7.19 (1 H, dt, *H*-8, J_t 7.8, J_d 1.5). ^{13}C NMR (75 MHz, $CDCl_3$, 25 °C) δ 14.2 (CH_3CH_2); 41.4 (CH_2CO_2Et); 46.4 (*C*-5); 51.8 (*C*NH); 56.1 (x2) (OCH_3); 60.9 (OCH_3); 61.6 (*C*H-C=O); 71.5 (*C*-2); 106.0 (*C*H trimethoxyphenyl); 119.6 (*C*-9); 123.1 (*C*-7); 127.2 (*C*-5a); 129.1 (*C*-6); 129.5 (quat. trimethoxyphenyl); 129.7 (*C*-8); 137.9, 153.4 (*C*-OMe); 157.2 (*C*-9a); 169.3, 169.5, 170.1 (*C*=O). I.r. (ATR): ν_{max} 3299, 2958, 1755, 1746, 1685, 1654, 1598, 1548, 1511, 1490, 1468, 1452, 1432, 1352, 1332, 1288, 1256, 1192, 1130, 1112, 1098, 1082, 1062, 1019, 1000, 958, 922, 864, 810, 783, 757, 737, 717, 703, 678, 645, 623 cm^{-1}. GC-MS (initial temp. 100°C, final temp. 290°C): R_t 13.76 min; *m/z* 472 (M^+, 2.0%) 370 (12.1) 369 (49.6) 342 (19.2) 264 (16.6) 263 (100) 209 (9.8) 208 (60.2) 207 (17.1) 194 (7.2) 193 (26.6) 182 (7.7) 181 (16.1) 178 (10.8) 177 (38.1) 165 (5.6) 163 (6.5) 162 (14.1) 150 (9.6) 147 (8.7) 146 (18.3) 136 (5.3) 135 (7.7) 134 (6.8) 133 (7.4) 121 (5.8) 120 (10.9) 119 (9.5) 118 (8.2) 107 (16.9) 105 (5.4) 96 (7.1) 92 (5.5) 91 (35.6) 90 (6.8) 89 (7.7) 79 (11.6) 78 (12.9) 77 (13.1) 74 (6.6) 65 (7.8) 56 (7.9) 44 (57.7) 42 (8.5) 40 (50.3). HRMS (ESI+) m/z: $[M + H]^+$ Calcd for $C_{24}H_{29}N_2O_8$ 473.1924; Found 473.1918.

_N_-Butyl-2-(3-oxo-2,3-dihydrobenzo[_f_][1,4]oxazepin-4(5_H_)-yl)-2-phenylacetamide (10e)

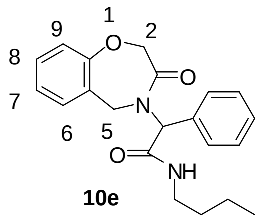

10e

White solid. M.p.: 100.2–101.8 °C. R_f 0.35 (PE/AcOEt 75:25). ^{1}H NMR (300 MHz, $CDCl_3$, 25 °C) δ 0.90 (3 H, t, CH_3CH$_2$, J 7.2); 1.23-1.37 (2 H, m, CH_2CH$_3$); 1.41-1.53 (2 H, m, CH_2CH$_2$CH$_3$); 3.20-3.43 (2 H, m, CH_2NH); 4.25 (1 H, d, *H*-5, J 15.3); 4.68 and 4.88 (2 H, AB syst., *H*-2, J_{AB} 15.6); 4.79 (1 H, d, *H*-5, J 15.3); 5.95 (1 H, broad t, N*H*); 6.38 (1 H, s, C*H*-C=O); 6.63 (1 H, dd, *H*-6, J 7.8, 1.5); 6.88 (1 H, dt, *H*-7, J_t 7.2, J_d 0.9); 6.99 (1 H, dd, *H*-9, J 8.1, 1.6); 7.20 (1 H, dt, *H*-8, J_t 8.1, J_d 1.5); 7.30-7.38 (5 H, m). ^{13}C NMR (75 MHz, $CDCl_3$, 25 °C) δ 13.7 (CH_3); 20.0 (CH_2CH_3); 31.3 ($CH_2CH_2CH_3$); 39.3 (CH_2NH); 45.7 (*C*-5); 60.4 (*C*H-C=O); 71.9 (*C*-2); 119.4 (*C*-9); 123.4 (*C*-7); 128.3 (*C*-5a); 128.4 (*C* para of Ph); 128.7, 128.9 (*C* ortho and meta of Ph); 129.0 (*C*-6); 129.5 (*C*-8); 134.8 (quat. of Ph); 157.3 (*C*-9a); 168.9, 169.7 (*C*=O). I.r. (ATR): ν_{max} 3282, 2956, 1666, 1645, 1558, 1491, 1474, 1451, 1430, 1364, 1339, 1307, 1262, 1234, 1219, 1199, 1173, 1156, 1115, 1065, 1043, 1025, 971, 953, 925, 862, 751, 735, 697, 648, 609 cm^{-1}. HRMS (ESI+) m/z: $[M + H]^+$ Calcd for $C_{21}H_{25}N_2O_3$ 353.1865; Found 353.1871.

N-Cyclohexyl-2-(2-methylpropyl)-2-(3-oxo-2,3-dihydrobenzo[f][1,4]oxazepin-4(5H)-yl)acetamide (10f)

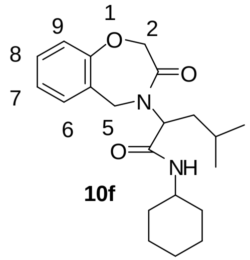

10f

White solid. M.p.: 94.8-95.7 °C. R_f 0.58 (PE/AcOEt 60:40).^{1}H NMR (300 MHz, $CDCl_3$, 25 °C) δ 0.77-0.96 (2 H, m); 0.886 (3 H, d, CH_3, J 6.6); 0.893 (3 H, d, CH_3, J 6.6); 1.00-1.87 (11 H, m); 3.64 (1 H, dtt, C*H*NH, J_t 3.7, 12.0, J_d 8.0); 4.51 and 4.59 (2 H, AB syst., *H*-5, J_{AB} 15.3); 4.75 and 4.79 (2 H, AB syst., *H*-2, J_{AB} 15.3); 5.13 (1 H, dd, C*H*-C=O, J 6.6, 9.0); 5.78 (1 H, d, N*H*, J 8.0); 6.99-7.06 (2 H, m, *H*-9, *H*-7); 7.16 (1 H, dd, *H*-6, J 1.6, 8.0); 7.27 (1 H, dt, *H*-8, J_d 1.6, J_t 7.7). ^{13}C NMR (75 MHz, $CDCl_3$, 25 °C) δ 22.0, 22.9 (*C*H_3); 24.5, 24.6, 25.3, 32.4, 32.6 (*C*H_2 of cyclohexyl); 24.7 (*C*H(CH_3)$_2$); 36.8 (*C*H_2-CH-C=O); 44.2 (*C*-5); 48.0 (*C*HNH); 54.8 (*C*H-C=O); 71.6 (*C*-2); 119.5 (*C*-9); 123.3 (*C*-7); 126.9 (*C*-5a); 129.1 (*C*-6); 129.9 (*C*-8); 157.2 (*C*-9a); 169.1, 169.6 (*C*=O). I.r. ($CHCl_3$): ν_{max} 3419, 2996, 2931, 2853, 1663, 1619, 1488, 1451, 1347, 1325, 1191, 1111 ,1055, 697 cm^{-1}. HRMS (ESI+) m/z: $[M + H]^+$ Calcd for $C_{21}H_{31}N_2O_3$ 359.2335; Found 359.2339.

2-Ethyl-2-(9-methoxy-3-oxo-2,3-dihydrobenzo[f][1,4]oxazepin-4(5H)-yl)-N-methylacetamide (10g)

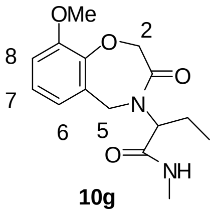

10g

White solid. M.p.: 163.6-166.0 °C. R_f 0.26 (PE/AcOEt 1:10). ^{1}H NMR (300 MHz, $CDCl_3$, 25 °C) δ 0.88 (3 H, t, CH_3CH_2, J 7.3); 1.78 (1 H, ddq, C*H*HCH_3, J_d 8.4, 14.5, J_q 7.3); 2.09 (1 H, d of quint, C*H*HC_3, J_d 14.5, J_{quint} 7.2); 2.73 (3 H, d, CH_3NH, J 4.8); 3.88 (3 H, s, OCH_3); 4.49 and 4.55 (2 H, AB syst., *H*-5, J_{AB} 14.9); 4.75 and 4.79 (2 H, AB syst., *H*-2, J_{AB} 16.0); 5.02 (1 H, dd, C*H*-C=O J 6.9, 8.4); 5.94 (1 H, broad s, N*H*); 6.80 (1 H, dd, *H*-8 or *H*-6, J 7.5, 1.5); 6.92 (1 H, dd, *H*-6 or *H*-8, J 8.1, 1.3); 7.03 (1 H, dd, *H*-7, J 7.5, 8.1). ^{13}C NMR (75 MHz, $CDCl_3$, 25 °C) δ 10.4 (*C*H_3CH_2); 21.4 (CH_3*C*H_2); 26.1 (*C*H_3NH); 43.5 (*C*-5); 56.0 (*C*H_3O); 58.2 (*C*H-C=O); 71.2 (*C*-2); 112.6, 120.3 (*C*-8, *C*-6); 124.4 (*C*-7); 130.8, 146.2, 150.9 (aromatic quat.); 170.0, 170.6 (*C*=O). I.r. (ATR): ν_{max} 3295, 2965, 1677, 1636, 1582, 1563, 1480, 1435, 1417, 1386, 1365, 1337, 1302, 1266, 1240, 1215, 1197, 1183, 1162, 1092, 1081, 1052, 1028, 990, 955, 782, 766, 751, 735, 689, 630 cm^{-1}. GC-MS: R_t 11.62 min; *m/z* 292 (M^+, 1.1%) 262 (13.3) 234 (6.0) 192 (31.5) 164 (5.3) 137 (7.4) 101 (8.0) 86 (15.4) 77 (7.6) 71 (5.6) 70 (100) 65 (8.7) 58 (17.0) 42 (13.0) 41 (9.3) 39 (5.1). HRMS (ESI+) m/z: $[M + Na]^+$ Calcd for $C_{15}H_{20}N_2NaO_4$ 315.1321; Found 315.1315.

N-Benzyl-2-(*tert*-butyl)-2-(9-methoxy-3-oxo-2,3-dihydrobenzo[*f*][1,4]oxazepin-4(5*H*)-yl)acetamide (10h)

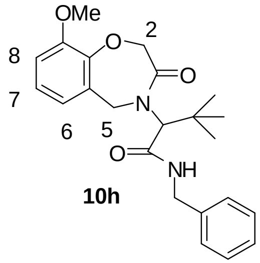

White solid. M.p.: 157.8-160.1 °C. R_f 0.53 (PE/AcOEt 66:34). ^{1}H NMR (300 MHz, $CDCl_3$, 25 °C) δ 1.11 (9 H,s, C(CH_3)$_3$); 3.88 (3 H, s, OCH_3); 4.23 and 4.44 (2 H, AB part of an ABX syst., CH_2Ph, J_{AB} 14.7, J_{AX} 5.4, J_{BX} 6.0); 4.69 (1 H, d, *H*-5, J 15.3); 4.88 (1 H, d, *H*-5, J 15.3); 4.87 and 4.94 (2 H, AB syst., *H*-2, J_{AB} 16.0); 4.97 (1 H, s, C*H*-C=O); 6.16 (1 H, t, N*H*, J 5.7); 6.79 (1 H, dd, *H*-8 or *H*-6, J 7.5, 1.8); 6.89 (1 H, dd, *H*-6 or *H*-8, J 8.2, 1.6); 6.96 (1 H, dd, *H*-7, J 7.5, 8.2); 7.09-7.15 (2 H, m); 7.22-7.30 (3 H, m). ^{13}C NMR (75 MHz, $CDCl_3$, 25 °C) δ 27.6 (C(CH_3)$_3$); 35.7 (*C*(CH_3)$_3$); 43.3 (*C*H_2Ph); 45.8 (*C*-5); 56.0 (*C*H_3O); 62.7 (*C*H-C=O); 71.8 (*C*-2); 112.2, 121.2 (*C*-8, *C*-6); 123.6 (*C*-7); 127.5, 127.7 (x2), 128.7 (*C*H benzyl); 129.3, 137.7, 146.4, 150.5 (aromatic quat.); 169.0, 170.2 (*C*=O). I.r. (ATR): ν_{max} 3332, 2970, 1673, 1621, 1543, 1492, 1464, 1424, 1366, 1336, 1311, 1292, 1270, 1207, 1184, 1080, 1065, 1054, 1044, 1031, 785, 754, 730, 700, 650, 632 cm^{-1}. GC-MS: R_t 15.01 min; *m*/*z* 396 (M^+, 0.9%) 290 (10.1) 289 (56.1) 262 (20.2) 233 (13.7) 205 (10.4) 193 (6.5) 192 (54.0) 191 (7.0) 190 (42.9) 178 (5.5) 164 (7.4) 162 (6.1) 149 (12.3) 137 (16.7) 136 (25.0) 135 (9.5) 107 (13.3) 106 (13.3) 105 (8.0) 98 (40.4) 92 (8.4) 91 (100) 86 (9.4) 79 (9.0) 78 (6.2) 77 (13.4) 69 (18.9) 65 (17.2) 57 (8.3) 56 (10.8) 51 (6.1) 42 (12.4) 41 (18.0) 39 (6.0). HRMS (ESI+) m/z: [M + Na]$^+$ Calcd for $C_{23}H_{28}N_2NaO_4$ 419.1947; Found 419.1951.

N-(*tert*-Butyl)-2-(9-methoxy-3-oxo-2,3-dihydrobenzo[*f*][1,4]oxazepin-4(5*H*)-yl)-2-phenylacetamide (10i)

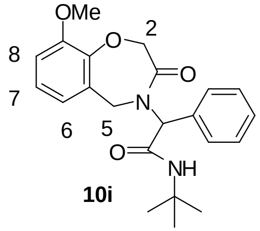

White solid. M.p.: 180.8-181.5 °C. R_f 0.29 (PE/AcOEt 66:34). ^{1}H NMR (300 MHz, $CDCl_3$, 25 °C) δ 1.37 (9 H, s, C(CH_3)$_3$); 3.85 (3 H, s, OCH_3); 4.17 (1 H, d, *H*-5, J 15.0); 4.68 and 4.94 (2 H, AB syst., *H*-2, J_{AB} 16.0); 4.84 (1 H, d, *H*-5, J 15.0); 5.64 (1 H, s, N*H*); 6.12 (1 H, m, *H*-6); 6.31 (1 H, s, C*H*-C=O); 6.78-6.86 (2 H, m, *H*-7 and *H*-8); 7.30-7.40 (5 H, m). ^{13}C NMR (75 MHz, $CDCl_3$, 25 °C) δ 28.7 (C(*C*H_3)$_3$); 45.1 (*C*-5); 51.9 (*C*NH); 55.9 (O*C*H_3); 60.9 (*C*H-C=O); 72.2 (*C*-2); 112.2 (*C*-8); 120.4 (*C*-6); 123.9 (*C*-7); 128.5, 128.8 (x2), 129.2 (x2) (*C*H of phenyl); 131.3 (*C*-5a); 135.1 (quat. of phenyl); 146.3, 150.7 (*C*-9, *C*-9a); 168.4, 169.7 (*C*=O). I.r. (ATR): ν_{max} 3298, 2957, 1684, 1610, 1548, 1490, 1481, 1472, 1455, 1434, 1419, 1363, 1314, 1288, 1276, 1266, 1224, 1209, 1187, 1098, 1083, 1062, 1033, 952, 821, 783, 751, 718, 702, 679, 646, 623 cm^{-1}. GC-MS: R_t 13.94 min; *m*/*z* 382 (M^+, 0.5%) 283 (10.0) 282 (14.6) 193 (7.1) 192 (64.3) 191 (78.3) 178 (7.1) 164 (6.7) 151 (19.0) 147 (6.2) 146 (11.9) 138 (8.6)

137 (17.5) 136 (19.7) 135 (39.5) 119 (15.6) 118 (100) 117 (5.3) 107 (10.2) 106 (18.8) 105 (8.1) 104 (11.2) 92 (10.6) 91 (85.0) 90 (10.4) 89 (6.5) 79 (9.6) 78 (6.6) 77 (16.1) 65 (14.4) 57 (24.4) 51 (6.3) 42 (5.6) 41 (14.7) 39 (7.0). HRMS (ESI+) m/z: $[M + H]^+$ Calcd for $C_{22}H_{27}N_2O_4$ 383.1971; Found 383.1976.

***N*-(2,6-Dimethylphenyl)-2-(9-methoxy-3-oxo-2,3-dihydrobenzo[*f*][1,4]oxazepin-4(*5H*)-yl)-2-phenylacetamide (10j)**

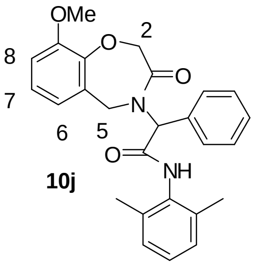

10j

White solid. M.p.: 176.0-180.0 °C. R_f 0.26 (PE/AcOEt 50:50). ^{1}H NMR (300 MHz, $CDCl_3$, 25 °C) δ 2.16 (6 H, s, C*H*$_3$Ar); 3.85 (3 H, s, OC*H*$_3$); 4.32 (1 H, d, *H*-5, J 14.7); 4.73 and 4.84 (2 H, AB syst., *H*-2, J_{AB} 16.2); 4.77 (1 H, d, *H*-5, J 15.0); 6.28 (1 H, apparent dd, *H*-6, J 3.3, 6.0); 6.64 (1 H, s, C*H*-C=O); 6.80-6.90 (2 H, m, *H*-7 and *H*-8); 7.00-7.12 (3 H, m); 7.37-7.45 (3 H, m); 7.47-7.53 (2 H, m); 7.61 (1 H, s, N*H*). ^{13}C NMR (75 MHz, $CDCl_3$, 25 °C) δ 18.4 (Ar*C*H$_3$); 45.0 (*C*-5); 55.9 (O*C*H$_3$); 60.7 (*C*H-C=O); 72.3 (*C*-2); 112.3 (*C*-8); 120.6 (*C*-6); 124.2 (*C*-7); 127.5, 128.2 (x2), 128.7, 128.9 (x2), 129.2 (x2) (*C*H of phenyl and dimethylphenyl); 131.4 (*C*-5a); 133.2, 134.3, 135.4 (x2) (quat. of phenyl and dimethylphenyl); 146.3, 150.7 (*C*-9, *C*-9a); 167.6, 170.1 (*C*=O). I.r. (ATR): ν_{max} 3253, 2924, 1668, 1615, 1489, 1467, 1377, 1310, 1267, 1206, 1163, 1079, 1052, 958, 918, 824, 768, 720, 698 cm^{-1}. GC-MS (initial temp. 100°C, final temp. 290°C): R_t 14.26 min; *m/z* 430 (M^+, 0.5%) 310 (9.5) 309 (6.0) 282 (17.0) 240 (13.2) 239 (69.6) 193 (5.6) 192 (40.8) 164 (5.4) 151 (7.1) 149 (7.4) 148 (48.7) 147 (5.5) 146 (9.1) 137 (14.1) 136 (14.0) 135 (9.5) 132 (5.1) 121 (10.2) 120 (9.7) 119 (14.3) 118 (95.7) 117 (5.8) 107 (10.4) 106 (15.8) 105 (14.7) 104 (11.5) 92 (11.8) 91 (100) 90 (12.6) 89 (7.1) 79 (10.6) 78 (8.8) 77 (25.2) 65 (15.1) 51 (9.1) 44 (9.2) 40 (7.9) 39 (7.2). HRMS (ESI+) m/z: $[M + H]^+$ Calcd for $C_{26}H_{27}N_2O_4$ 431.1971; Found 431.1973.

***N*-(*tert*-Butyl)-2-cyclohexyl-2-(3-oxo-7-phenyl-2,3-dihydrobenzo[*f*][1,4]oxazepin-4(*5H*)-yl)acetamide (10k)**

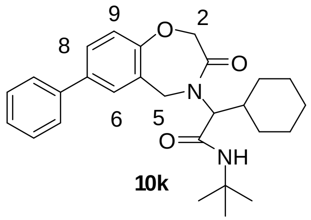

10k

White solid. M.p.: 135.2-136.0 °C. R_f 0.61 (PE/AcOEt 66:34). ^{1}H NMR (300 MHz, $CDCl_3$, 25 °C) δ 0.80-1.04 (3 H, m); 1.18 (9 H, s, C(C*H*$_3$)$_3$); 1.10-1.38 (2 H, m); 1.45-1.85 (5 H, m); 2.10 (1 H, tq, C*H*CH-C=O, J_t 3.3, J_q 11.1); 4.58 (1 H, d, C*H*-C=O, J 11.1); 4.68 and 4.81 (2 H, AB syst., *H*-5, J_{AB} 15.4); 4.75 and 4.89 (2 H, AB syst., *H*-2, J_{AB} 15.0); 5.68 (1 H, s, N*H*); 7.04 (1 H, d, *H*-9, J 8.1); 7.32 (1 H, dt, *H*-8, J_d 7.2, J_t 1.3); 7.38-7.55 (5

H, m). ^{13}C NMR (75 MHz, $CDCl_3$, 25 °C) δ 25.5, 25.6, 26.3, 28.9, 29.8 (cyclohexyl *C*H_2); 28.4 (C(*C*H_3)$_3$); 35.6 (cyclohexyl *C*H); 44.3 (*C*-5); 51.4 (*C*NH); 62.0 (*C*H-C=O); 71.5 (*C*-2); 119.8 (*C*-9); 126.8 (x2), 126.9, 127.2, 128.3 (x2), 128.8 (x2) (other aromatic *C*H and *C*-5a); 136.3, 140.0, 156.7 (quat.); 168.6, 169.6 (*C*=O). I.r. (ATR): ν_{max} 3328, 2927, 1647, 1622, 1544, 1510, 1484, 1451, 1406, 1391, 1363, 1329, 1258, 1226, 1193, 1178, 1123, 1063, 1023, 892, 826, 761, 718, 697, 651 cm^{-1}. GC-MS: R_t 13.33 min; *m*/*z* 238 (M^+-196, 3.4%) 197 (15.7) 182 (5.9) 165 (5.7) 154 (8.4) 153 (10.6) 152 (9.5) 141 (6.9) 128 (6.2) 124 (9.3) 115 (15.7) 112 (10.7) 111 (6.0) 110 (5.8) 98 (16.6) 96 (9.4) 95 (84.6) 94 (6.0) 93 (11.2) 91 (5.2) 85 (11.2) 82 (6.0) 81 (10.8) 80 (7.7) 79 (6.5) 77 (11.3) 68 (11.2) 67 (32.1) 59 (8.6) 58 (17.7) 57 (61.3) 56 (17.1) 55 (37.2) 54 (5.8) 53 (8.9) 44 (19.8) 43 (15.2) 42 (100) 41 (63.0) 40 (10.7) 39 (9.7). HRMS (ESI+) m/z: $[M + H]^+$ Calcd for $C_{27}H_{35}N_2O_3$ 435.2648; Found 435.2649.

_N_-(2,6-Dimethylphenyl)-2-isobutil-2-(3-oxo-7-phenyl-2,3-dihydrobenzo[_f_][1,4]oxazepin-4(5_H_)-yl)acetamide (10l)

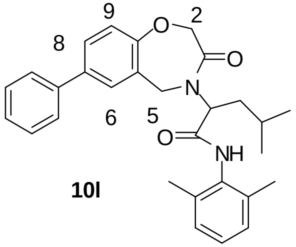

10l

White solid. M.p.: 165.3-167.7 °C. R_f 0.41 (PE/AcOEt 75:25). ^{1}H NMR (300 MHz, $CDCl_3$, 25 °C) δ 0.97 (6 H, d, C*H*$_3$CH, J 6.6); 1.55-1.67 (1 H, m, C*H*(CH_3)$_2$); 1.82 (1 H, ddd, C*H*H-*i*Pr, J 6.0, 8.1, 14.1); 2.01 (1 H, dt, C*H*H-*i*Pr, J_t 7.5, J_d 14.1); 2.01 (6 H, s, C*H*$_3$Ar); 4.71 (1 H, s, *H*-5); 4.82 and 4.87 (2 H, AB syst., *H*-2, J_{AB} 15.3); 5.40 (1 H, t, C*H*-C=O, J 7.5); 6.96-7.10 (3 H, m); 7.11 (1 H, d, *H*-9, J 8.4); 7.28-7.46 (6 H, m); 7.50 (1 H, dd, *H*-8, J 2.1, 8.4). ^{13}C NMR (75 MHz, $CDCl_3$, 25 °C) δ 18.1 (*C*H_3Ar); 22.4, 22.8 (*C*H_3CH); 25.1 (*C*H(CH_3)$_2$); 36.7 (*C*H_2*i*Pr); 44.5 (*C*-5); 54.9 (*C*H-C=O); 72.0(*C*-2); 120.1 (*C*-9); 126.9 (x2), 127.3 (x2), 127.6, 127.9, 128.1 (x2), 128.8 (x2) (other aromatic *C*H); 128.7, 133.3, 135.0 (x2), 137.1, 140.0, 156.9 (aromatic quat.); 168.5, 170.2 (*C*=O). I.r. (ATR): νmax 3255, 3028, 2961, 2916, 2872, 1663, 1645, 1524, 1512, 1481, 1466, 1445, 1431, 1403, 1379, 1365, 1331, 1282, 1238, 1219, 1208, 1187, 1169, 1130, 1121, 1070, 1046, 1027, 1021, 944, 827, 790, 768, 760, 711, 694, 669 cm–1. GC-MS: R_t 9.50 min; *m*/*z* 336 (M^+-120, 2.0%) 253 (23.0) 252 (6.0) 251 (72.1) 211 (5.9) 209 (18.4) 181 (14.0) 126 (8.8) 125 (7.4) 124 (26.3) 123 (5.6) 89 (43.0) 63 (16.2) 62 (7.9) 41 (100) 39 (19.3). HRMS (ESI+) m/z: $[M + H]^+$ Calcd for $C_{29}H_{33}N_2O_3$ 457.2491; Found 457.2488.

N-Butyl-2-(3-oxo-2,3-dihydronaphtho[2,3-*f*][1,4]oxazepin-4(*5H*)-yl)-2-(3,4,5-trimethoxyphenyl)acetamide (10m)

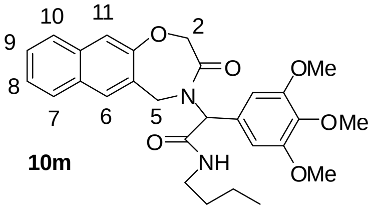

10m

White solid. M.p.: 65.2-70.5 °C. R_f 0.34 (PE/AcOEt 43:57). ^{1}H NMR (300 MHz, $CDCl_3$, 25 °C) δ 0.88 (3 H, t, C*H*$_3$CH$_2$, J 7.2); 1.32 (2 H, hexuplet, CH$_2$C*H*$_2$CH$_3$, J 7.4); 1.43-1.56 (2 H, m, C*H*$_2$CH$_2$NH); 3.22-3.47 (2 H, m, C*H*$_2$NH); 3.68 (6 H, s, C*H*$_3$O); 3.87 (3 H, s, C*H*$_3$O); 4.42 (1 H, d, *H*-5, J 15.0); 4.74 and 4.97 (2 H, AB syst., *H*-2, J_{AB} 16.2); 4.99 (1 H, d, *H*-5, J 15.0); 5.95 (1 H, t, N*H*, J 5.5); 6.29 (1 H, s, C*H*-C=O); 6.56 (2 H, s, C*H* of trimethoxyphenyl); 7.18 (1 H, s, *H*-11 or *H*-6); 7.32-7.50 (2 H, m, *H*-8, *H*-9); 7.47 (1 H, s, *H*-6 or *H*-11); 7.65 (1 H, d, *H*-10 or *H*-7, J 8.1); 7.73 (1 H, d, *H*-10 or *H*-7, J 7.8). ^{13}C NMR (75 MHz, $CDCl_3$, 25 °C) δ 13.7 (*C*H$_3$CH$_2$); 20.0 (CH$_3$*C*H$_2$); 31.5 (*C*H$_2$CH$_2$NH); 39.4 (*C*H$_2$NH); 45.7 (*C*-5); 56.1 (x2), 60.9 (O*C*H$_3$); 60.9 (*C*H-C=O); 73.3 (*C*-2); 106.3 (*C*H trimethoxyphenyl); 116.3 (*C*-6 or *C*-11); 125.4 (*C*-8 or *C*-9); 126.7 (*C*-8 or *C*-9) 127.0, 127.7 (*C*-7 and *C*-10); 128.1 (*C*-6 or *C*-11); 129.8, 130.0, 130.3, 134.2, 138.2, 153.5 (x2), 155.3 (quat.); 168.8, 169.8 (*C*=O). I.r. (ATR): ν_{max} 3308, 2961, 1659, 1635, 1590, 1543, 1506, 1456, 1419, 1368, 1332, 1247, 1154, 1125, 1111, 1007, 926, 869, 796, 750, 733, 701, 673, 644, 619 cm^{-1}. HRMS (ESI+) m/z: $[M + H]^+$ Calcd for $C_{28}H_{33}N_2O_6$ 493.2339; Found 493.2334.

***N*-Benzyl-2-(*tert*-butyl)-2-(3-oxo-2,3-dihydronaphtho[2,3-*f*][1,4]oxazepin-4(*5H*)-yl)acetamide (10n)**

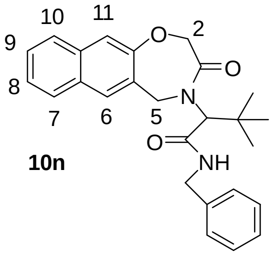

10n

White solid. M.p.: 145.8-148.4 °C. R_f 0.32 (PE/AcOEt 75:25). ^{1}H NMR (300 MHz, $CDCl_3$, 25 °C) δ 1.16 (9 H, s, C(C*H*$_3$)$_3$); 4.23 and 4.40 (2 H, AB part of an ABX syst., C*H*$_2$Ph, J_{AB} 14.6, J_{AX} 5.5, J_{BX} 6.0); 4.66 and 4.95 (2 H, AB syst., *H*-2, J_{AB} 15.6); 4.98 (2 H, s, C*H*-C=O); 5.10 (1 H, s, *H*-5); 6.10 (1 H, t, N*H*, J 5.7); 6.98-7.04 (2 H, m); 7.06-7.15 (3 H, m); 7.41 (1 H, dt, *H*-8 or *H*-9, J_d 1.2, J_t 7.5); 7.46 (1 H, s, *H*-6 or *H*-11); 7.41 (1 H, dt, *H*-8 or *H*-9, J_d 1.2, J_t 7.5); 7.74 (1 H, s, *H*-6 or *H*-11); 7.76 (1 H, d, *H*-7 or *H*-10, J 8.1); 7.78 (1 H, d, *H*-7 or *H*-10, J 7.8). ^{13}C NMR (75 MHz, $CDCl_3$, 25 °C) δ 27.7 (C(*C*H$_3$)$_3$); 35.8 (*C*(CH$_3$)$_3$); 43.3 (*C*H$_2$NH); 46.1 (*C*-5); 62.8 (*C*H-C=O); 72.8 (*C*-2); 115.9 (*C*-6 or *C*-11); 125.2 (*C*-8 or *C*-9); 126.8 (*C*-8 or *C*-9) 127.0, 127.4 (*C*-7 and *C*-10); 127.6 (x2), 128.6 (x2), 128.9 (*C*H benzyl); 128.0 (*C*-6 or *C*-11); 128.9, 130.3, 134.4, 137.5, 155.3 (quat.); 168.9, 170.3 (*C*=O). I.r. (ATR): ν_{max} 3299, 2959, 1646, 1616, 1541, 1507, 1467, 1340, 1242, 1158, 1112, 1039, 1016, 963, 874, 747, 698, 672, 619 cm^{-1}. GC-MS: R_t 13.66 min; *m/z* 416 (M^+, 2.6%) 310 (15.4) 309 (71.3) 282 (18.7) 281 (5.8) 253 (15.6) 213 (13.4) 212 (89.9) 205 (5.4) 198 (6.4) 197 (6.2) 191 (6.8) 190 (42.4) 184 (16.2) 183 (5.4) 182 (6.8) 170 (6.7) 169 (21.4) 157 (21.0) 156 (18.3) 142 (6.1) 141 (29.8) 139 (12.5) 129 (

8.9) 128 (39.2) 127 (10.8) 115 (10.8) 106 (9.2) 98 (41.2) 92 (8.2) 91 (100) 86 (10.9) 83 (6.9) 77 (6.3) 69 (24.7) 65 (9.4) 57 (10.1) 56 (13.1) 55 (5.6) 44 (9.8) 43 (5.9) 42 (9.7) 41 (23.7) 40 (9.0) 39 (5.3). HRMS (ESI+) m/z: $[M + H]^+$ Calcd for $C_{26}H_{29}N_2O_3$ 417.2178; Found 417.2179.

REFERENCES

[1] Lee, J.; Lee, J.-H.; Kim, S. Y.; Perry, N. A.; Lewin, N. E.; Ayres, J. A.; Blumberg, P. M. *Bioorg. Med. Chem.* **2006**, *14*, 2022-2031.

[2] Somu, R. V.; Boshoff, H.; Qiao, C.; Bennett, E. M.; Barry, C. E.; Aldrich, C. C. *J. Med. Chem.* **2005**, *49*, 31-34.

[3] Barluenga, J.; Tomas-Gamasa, M.; Valdes, C. *Angew. Chem., Int. Ed.* **2012**, *51*, 5950-5952.

[4] Nawrat, C. C.; Palmer, L. I.; Blake, A. J.; Moody, C. J. *J. Org. Chem.* **2013**, *78*, 5587-5603.

[5] a) Shetty, R.; Moffett, K. K. *Tetrahedron Lett.* **2006**, *47*, 8021-8024. b) DeNinno, M. P.; Masamune, H.; Scott, R. W., Patent: US2004/198693 A1, **2004**.

[6] Ahn, J. H.; Cho, S. Y.; Ha, J. D.; Chu, S. Y.; Jung, S. H.; Jung, Y. S.; Baek, J. Y.; Choi, I. K.; Shin, E. Y.; Kang, S. K.; Kim, S. S.; Cheon, H. G.; Yang, S.-D.; Choi, J.-K. *Bioorg. Med. Chem. Lett.* **2002**, *12*, 1941-1946.

[7] Lucas, S.; Heim, R.; Negri, M.; Antes, I.; Ries, C.; Schewe, K. E.; Bisi, A.; Gobbi, S.; Hartmann, R. W. *J. Med. Chem.* **2008**, *51*, 6138-6149.

2) ^{1}H AND ^{13}C NMR SPECTRA OF ALL NEW COMPOUNDS

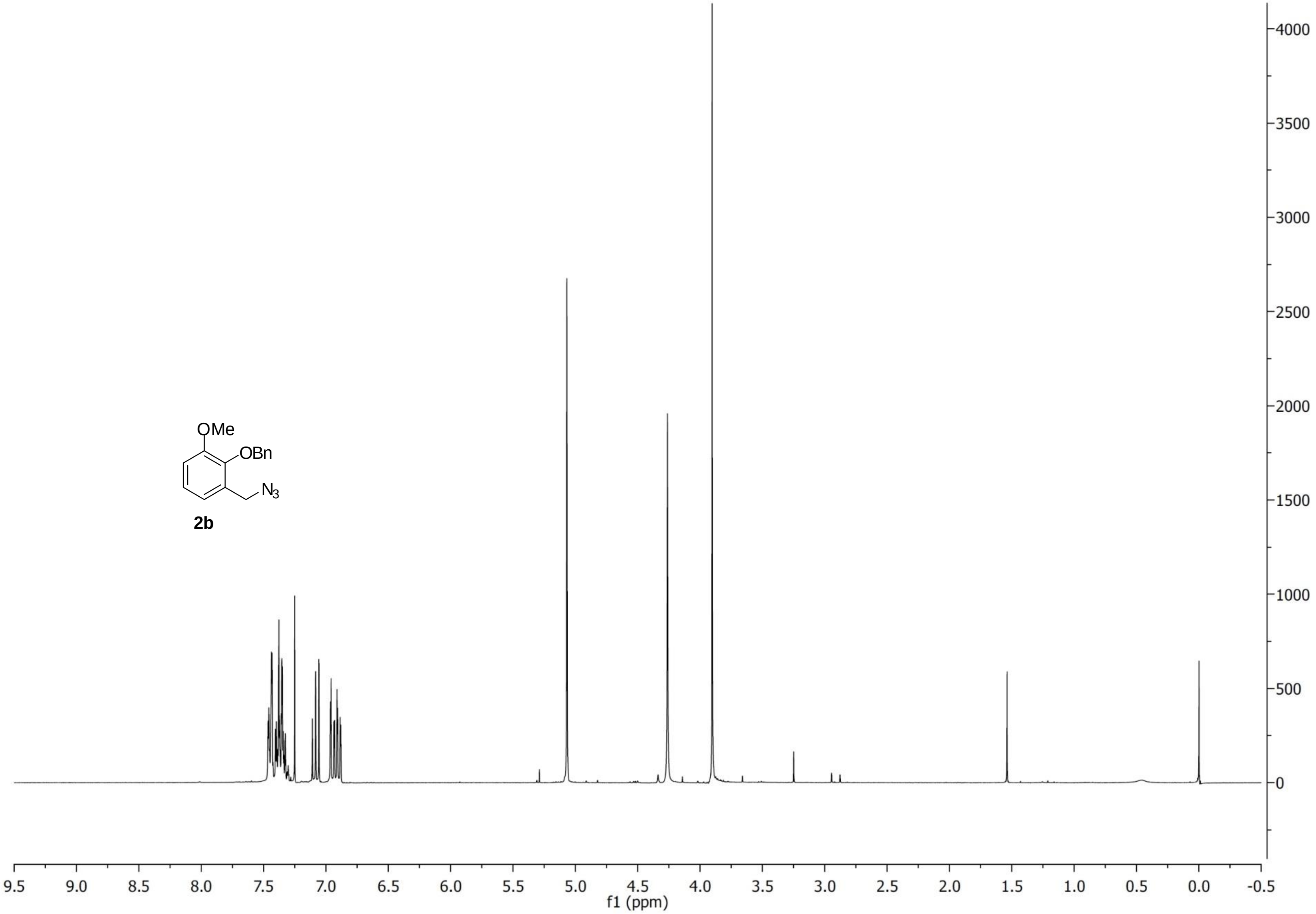

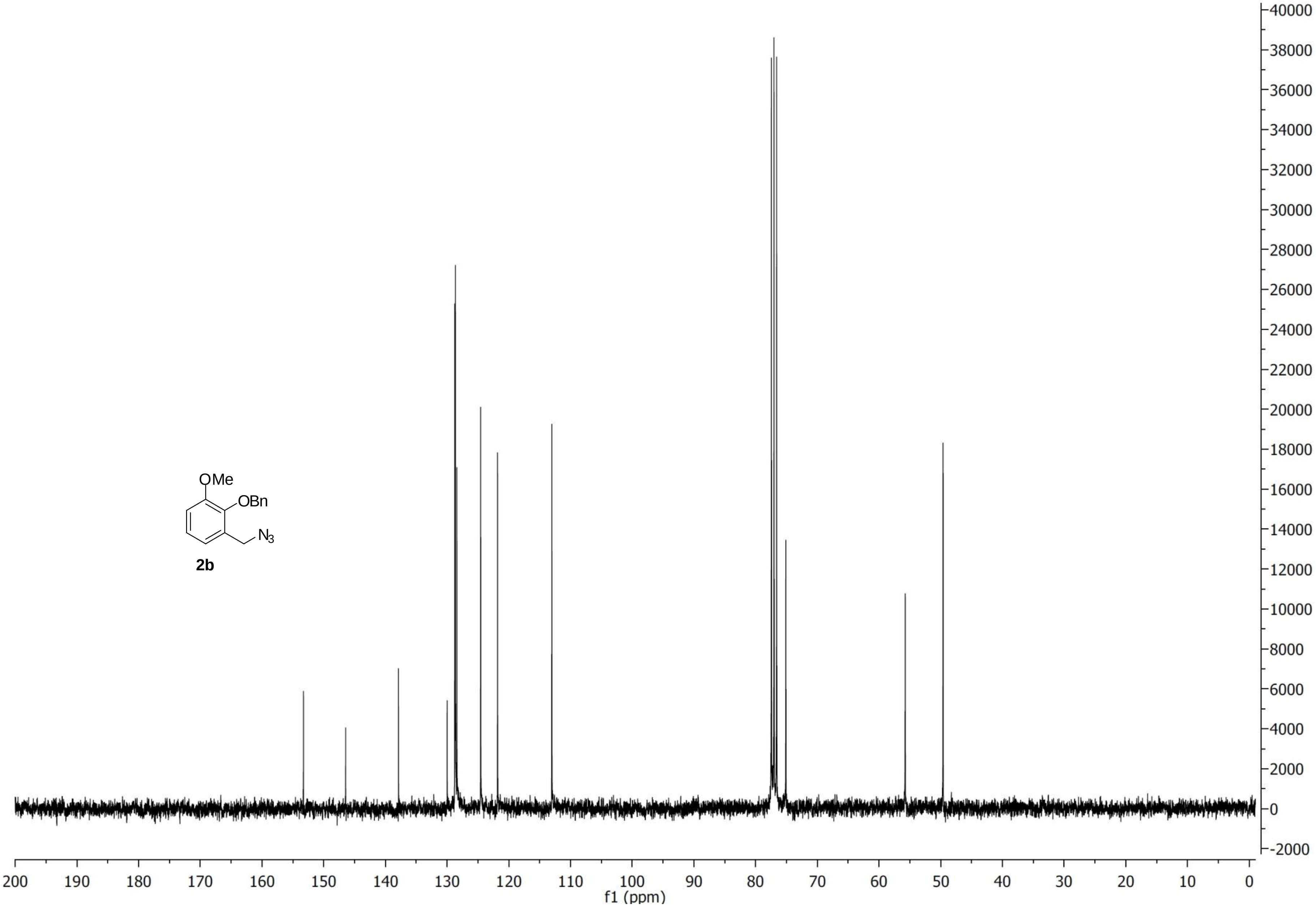
OMe
OBn
N3
2b
40000
38000
36000
34000
32000
30000
28000
26000
24000
22000
20000
18000
16000
14000
12000
10000
8000
6000
4000
2000
0
-2000
200 190 180 170 160 150 140 130 120 110 100 90 80 70 60 50 40 30 20 10 0
f1 (ppm)

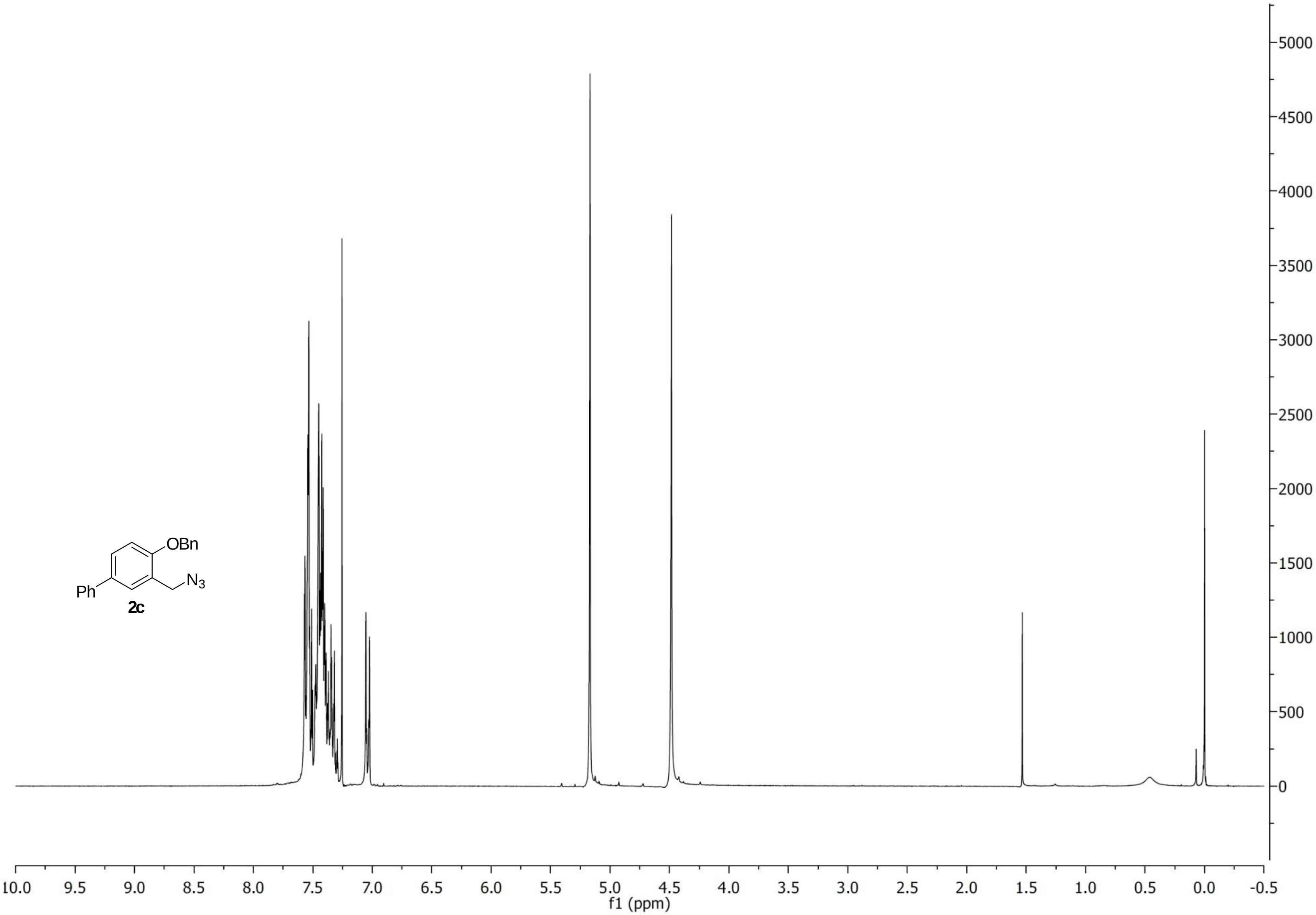
OBn
N3
Ph
2c
5000
4500
4000
3500
3000
2500
2000
1500
1000
500
0
10.0 9.5 9.0 8.5 8.0 7.5 7.0 6.5 6.0 5.5 5.0 4.5 4.0 3.5 3.0 2.5 2.0 1.5 1.0 0.5 0.0 -0.5
f1 (ppm)

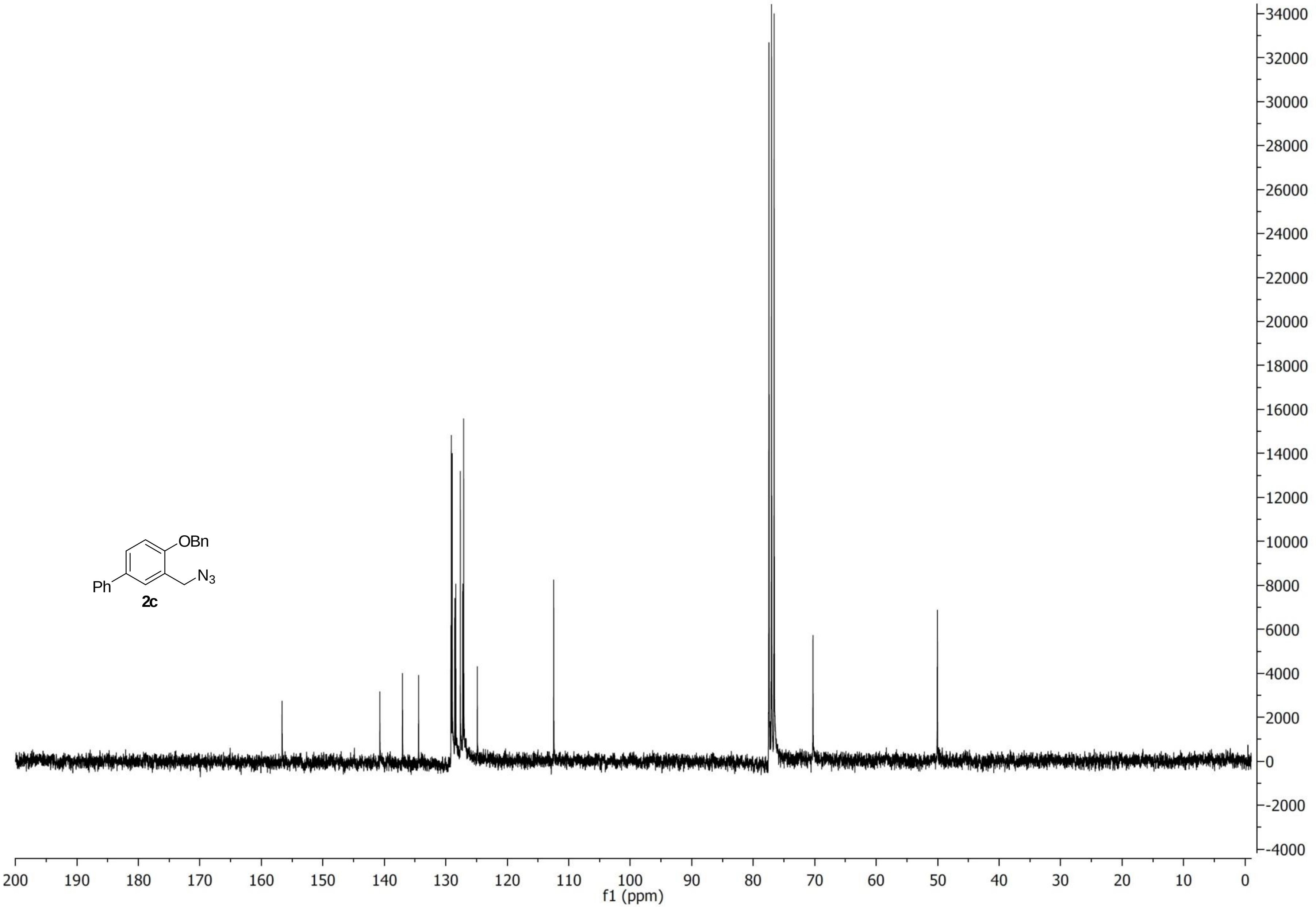
OBn
N3
Ph
2c
f1 (ppm)

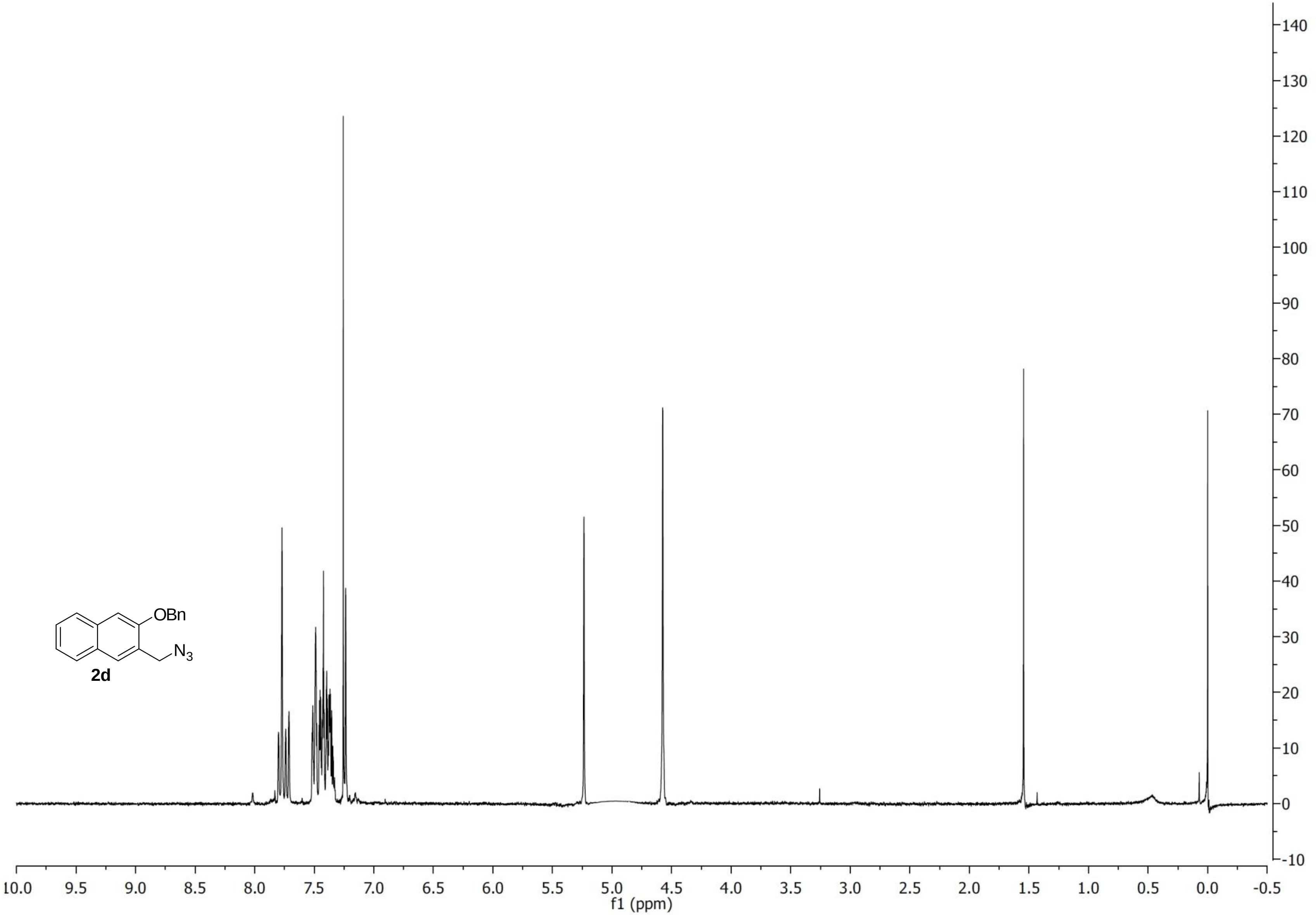
OBn
N3
2d
f1 (ppm)

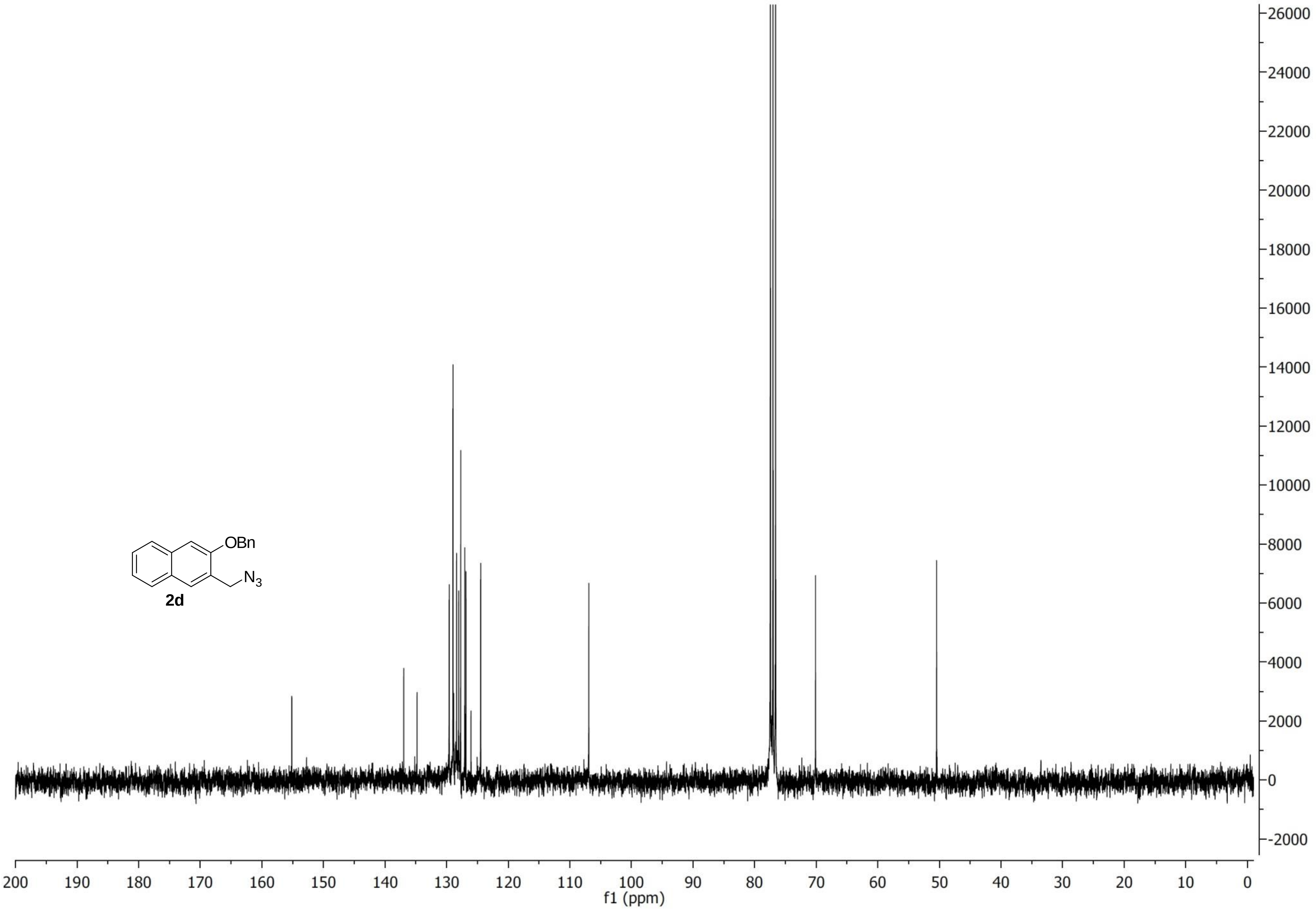
OBn
N_3
2d
f1 (ppm)
200
190
180
170
160
150
140
130
120
110
100
90
80
70
60
50
40
30
20
10
0
26000
24000
22000
20000
18000
16000
14000
12000
10000
8000
6000
4000
2000
0
-2000

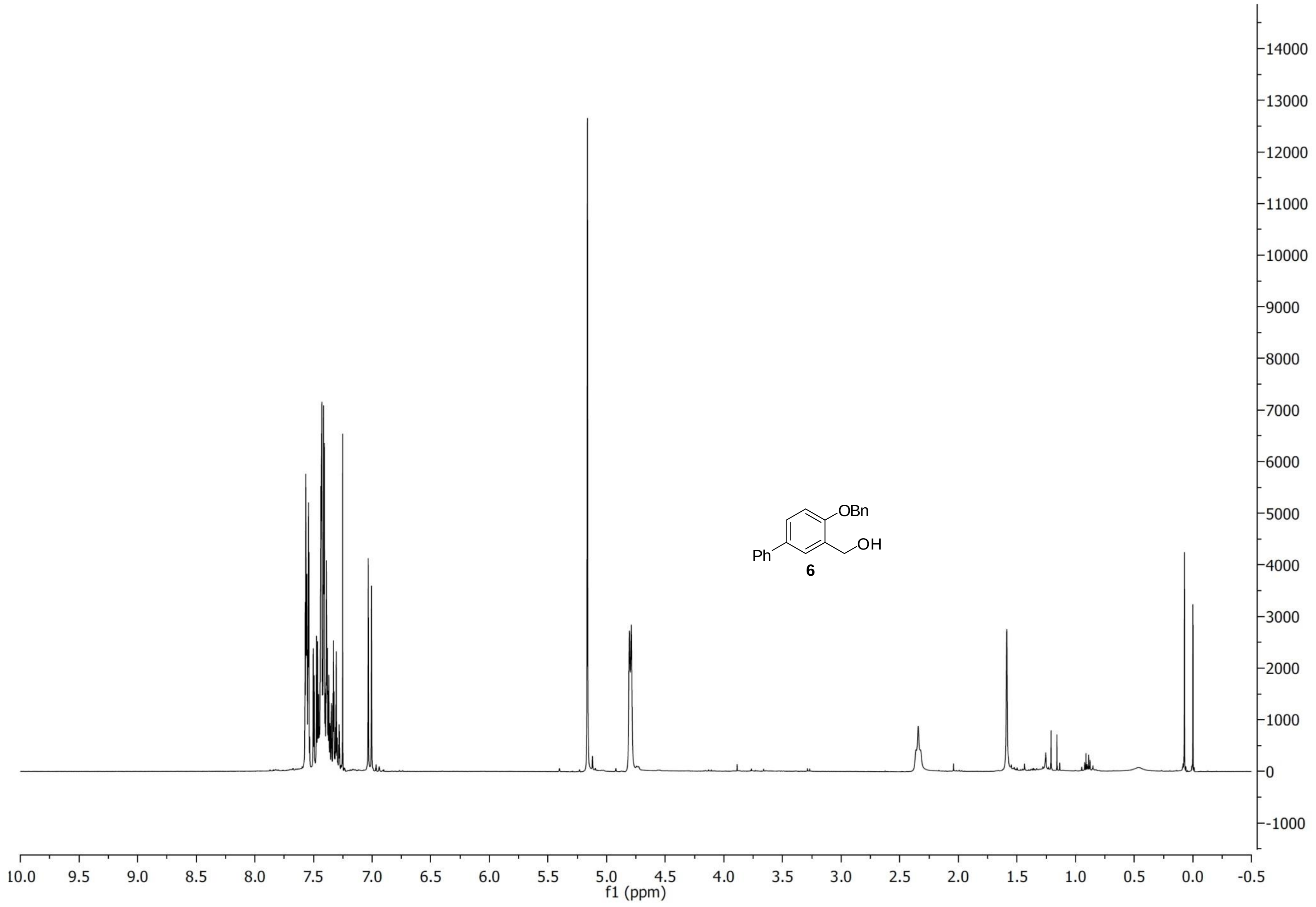
OBn
OH
Ph
6
14000
13000
12000
11000
10000
9000
8000
7000
6000
5000
4000
3000
2000
1000
0
-1000
10.0
9.5
9.0
8.5
8.0
7.5
7.0
6.5
6.0
5.5
5.0
4.5
4.0
3.5
3.0
2.5
2.0
1.5
1.0
0.5
0.0
-0.5
f1 (ppm)

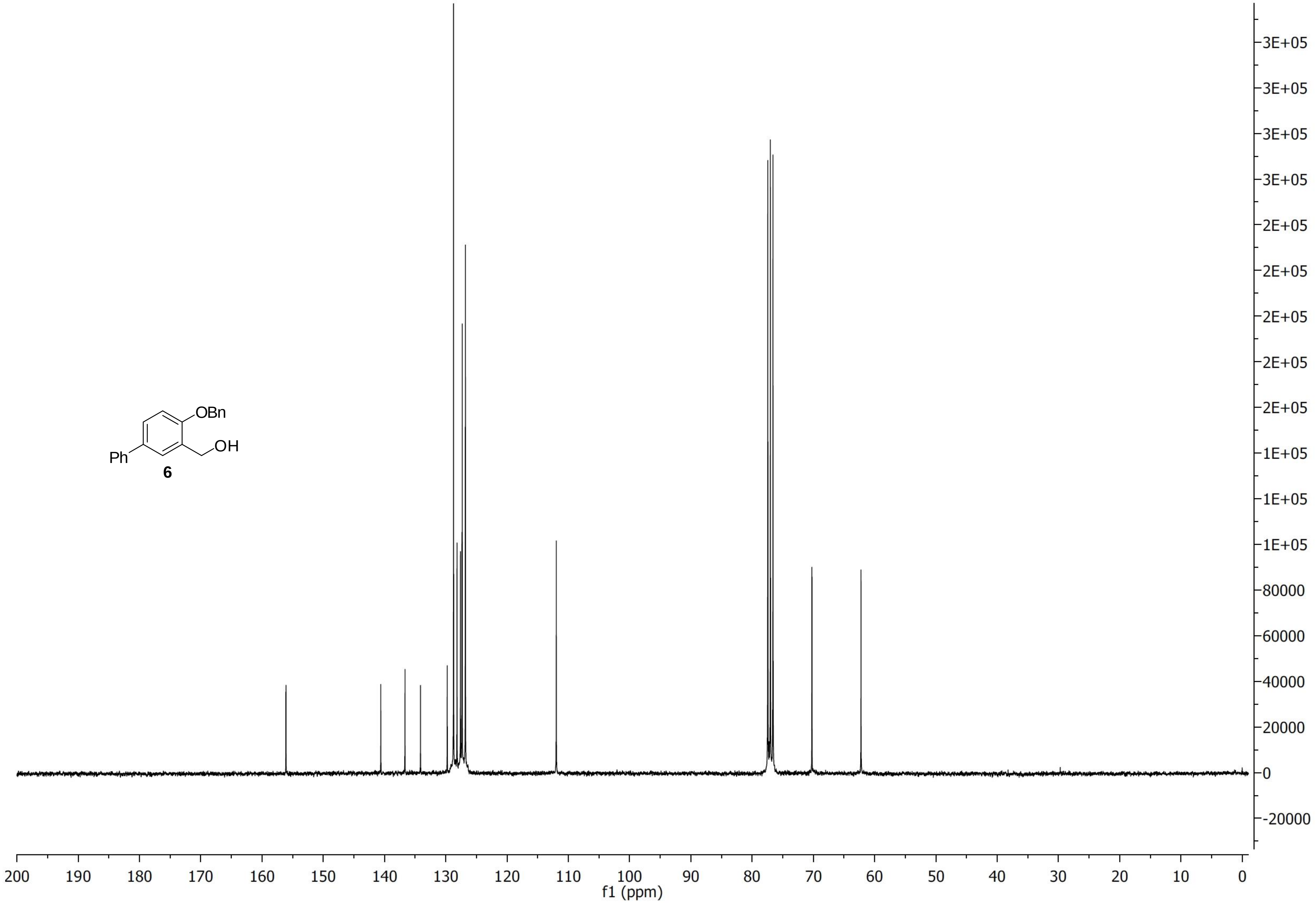
OBn
OH
Ph
6
3E+05
3E+05
3E+05
3E+05
2E+05
2E+05
2E+05
2E+05
2E+05
1E+05
1E+05
1E+05
80000
60000
40000
20000
0
-20000
200 190 180 170 160 150 140 130 120 110 100 90 80 70 60 50 40 30 20 10 0
f1 (ppm)

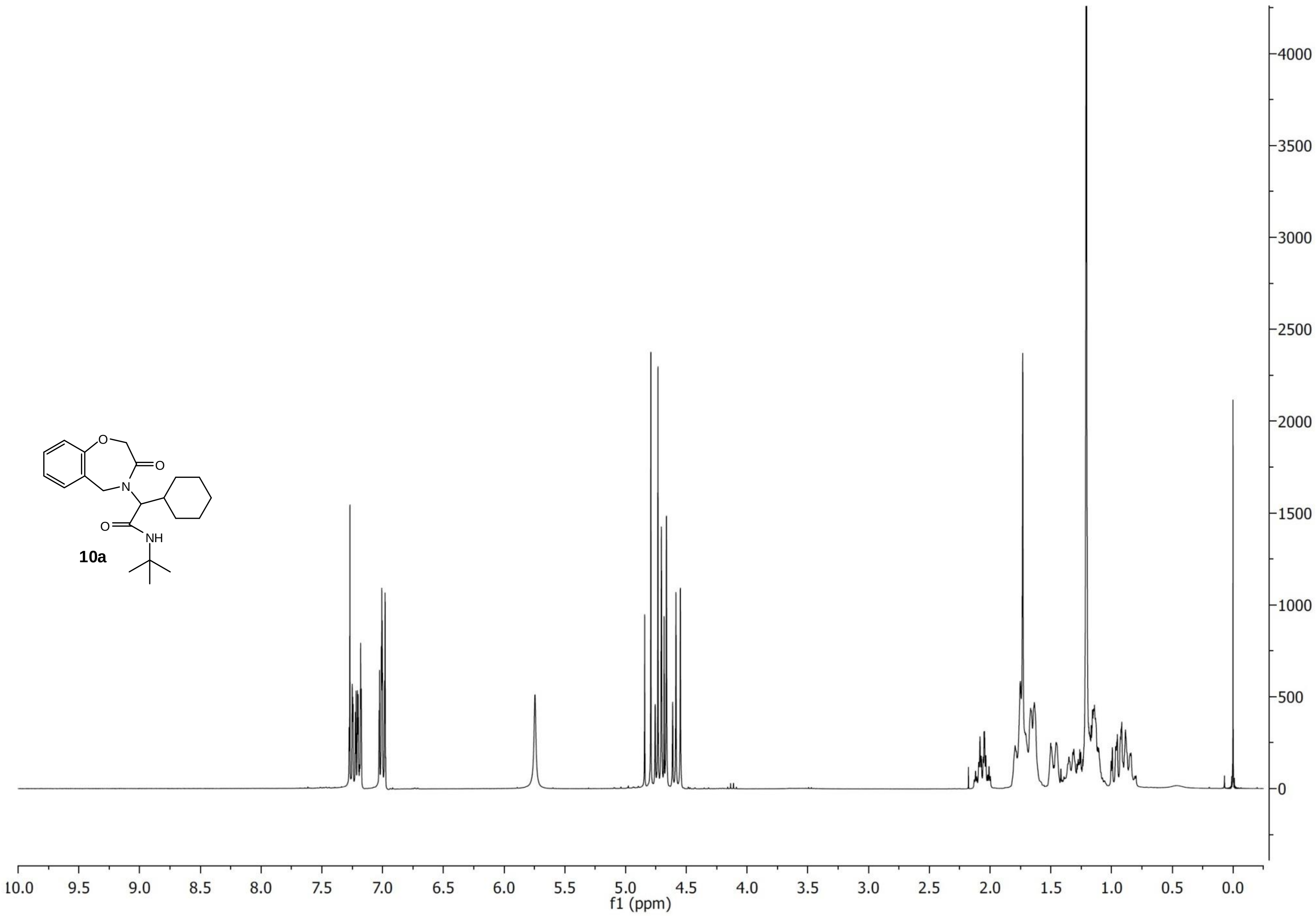
O
O
N
O
NH
10a
4000
3500
3000
2500
2000
1500
1000
500
0
10.0 9.5 9.0 8.5 8.0 7.5 7.0 6.5 6.0 5.5 5.0 4.5 4.0 3.5 3.0 2.5 2.0 1.5 1.0 0.5 0.0
f1 (ppm)

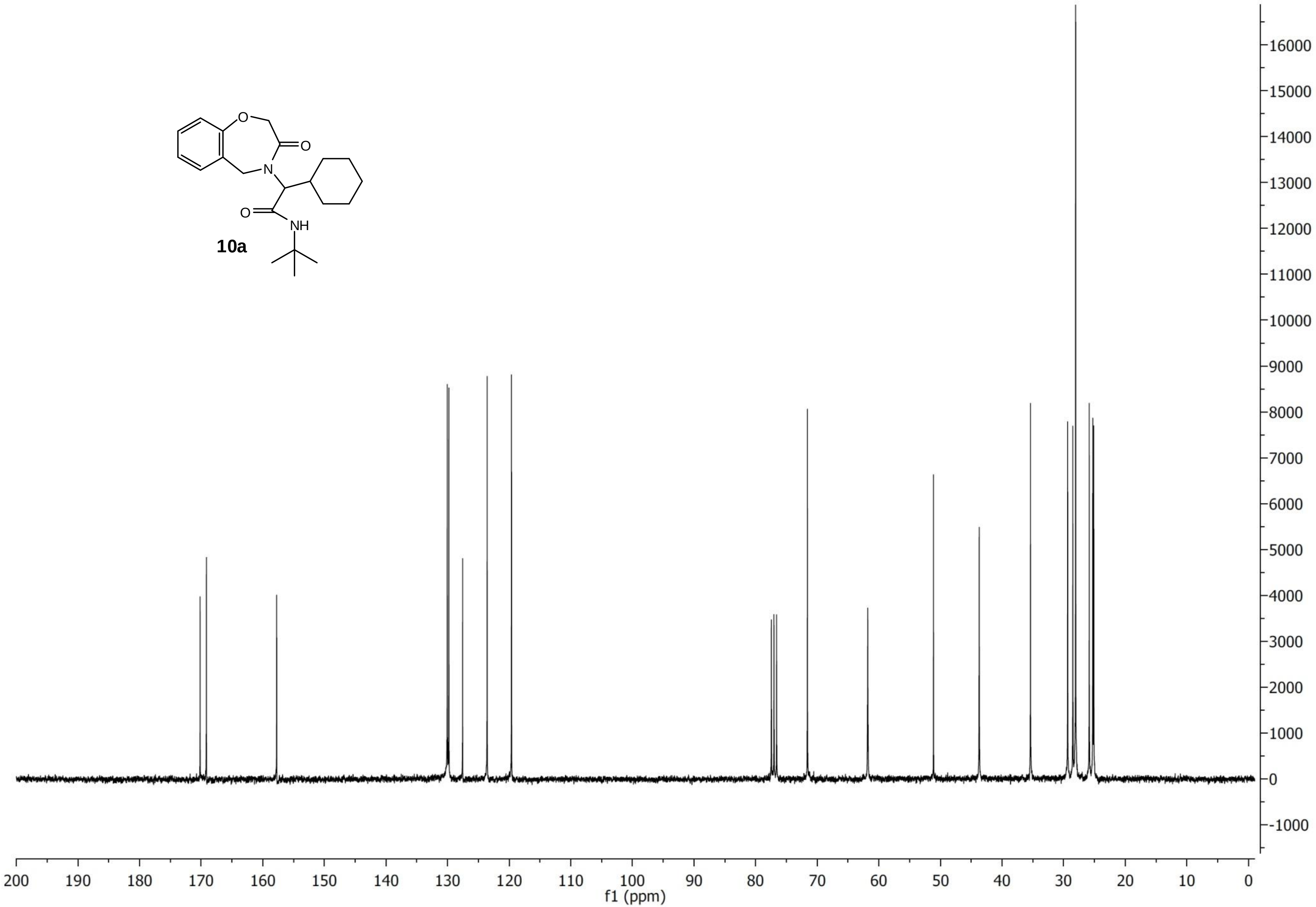
O
O
N
O
NH
10a
16000
15000
14000
13000
12000
11000
10000
9000
8000
7000
6000
5000
4000
3000
2000
1000
0
-1000
200 190 180 170 160 150 140 130 120 110 100 90 80 70 60 50 40 30 20 10 0
f1 (ppm)

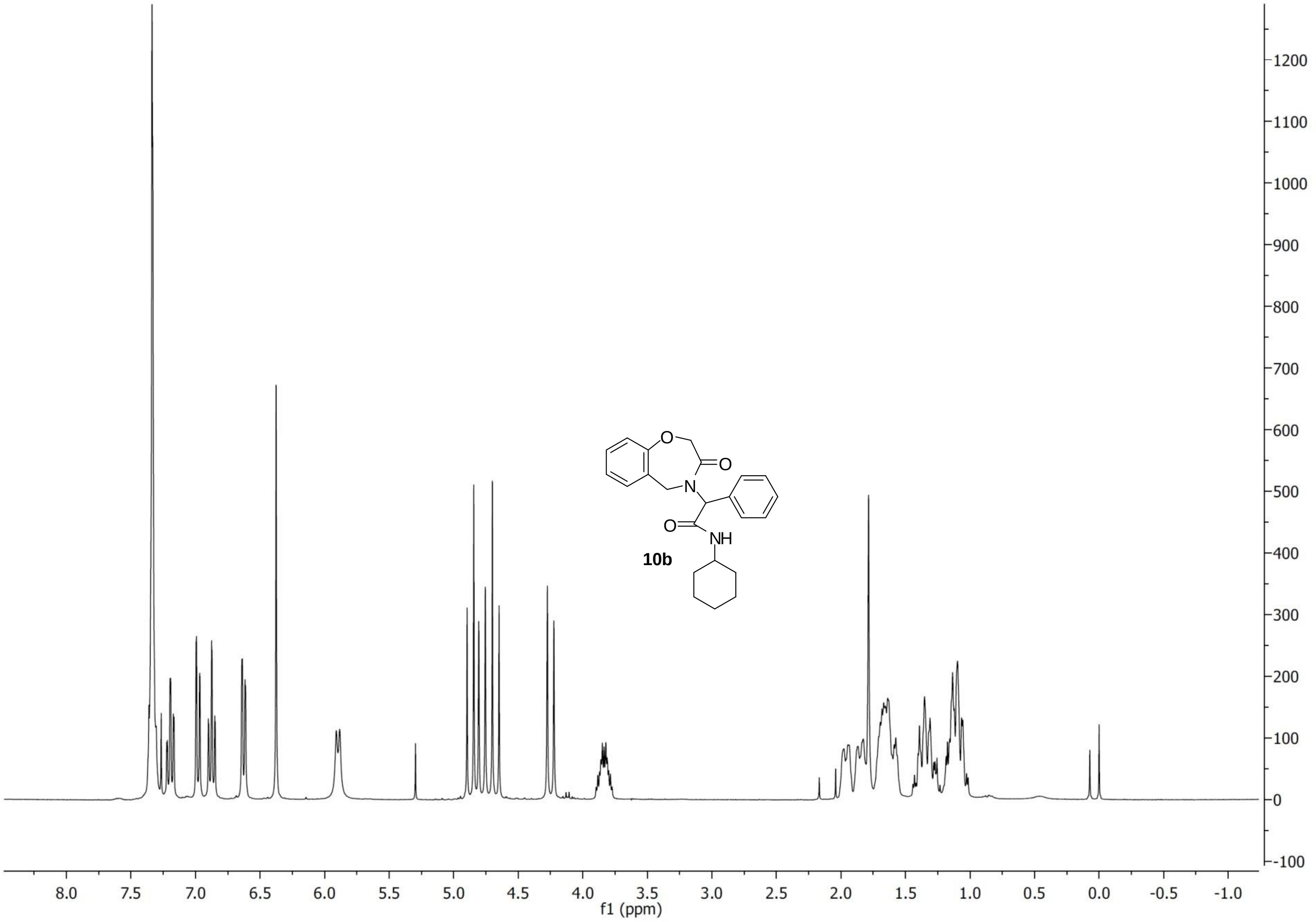
10b
O
N
O
O
NH
f1 (ppm)
8.0 7.5 7.0 6.5 6.0 5.5 5.0 4.5 4.0 3.5 3.0 2.5 2.0 1.5 1.0 0.5 0.0 -0.5 -1.0
1200 1100 1000 900 800 700 600 500 400 300 200 100 0 -100

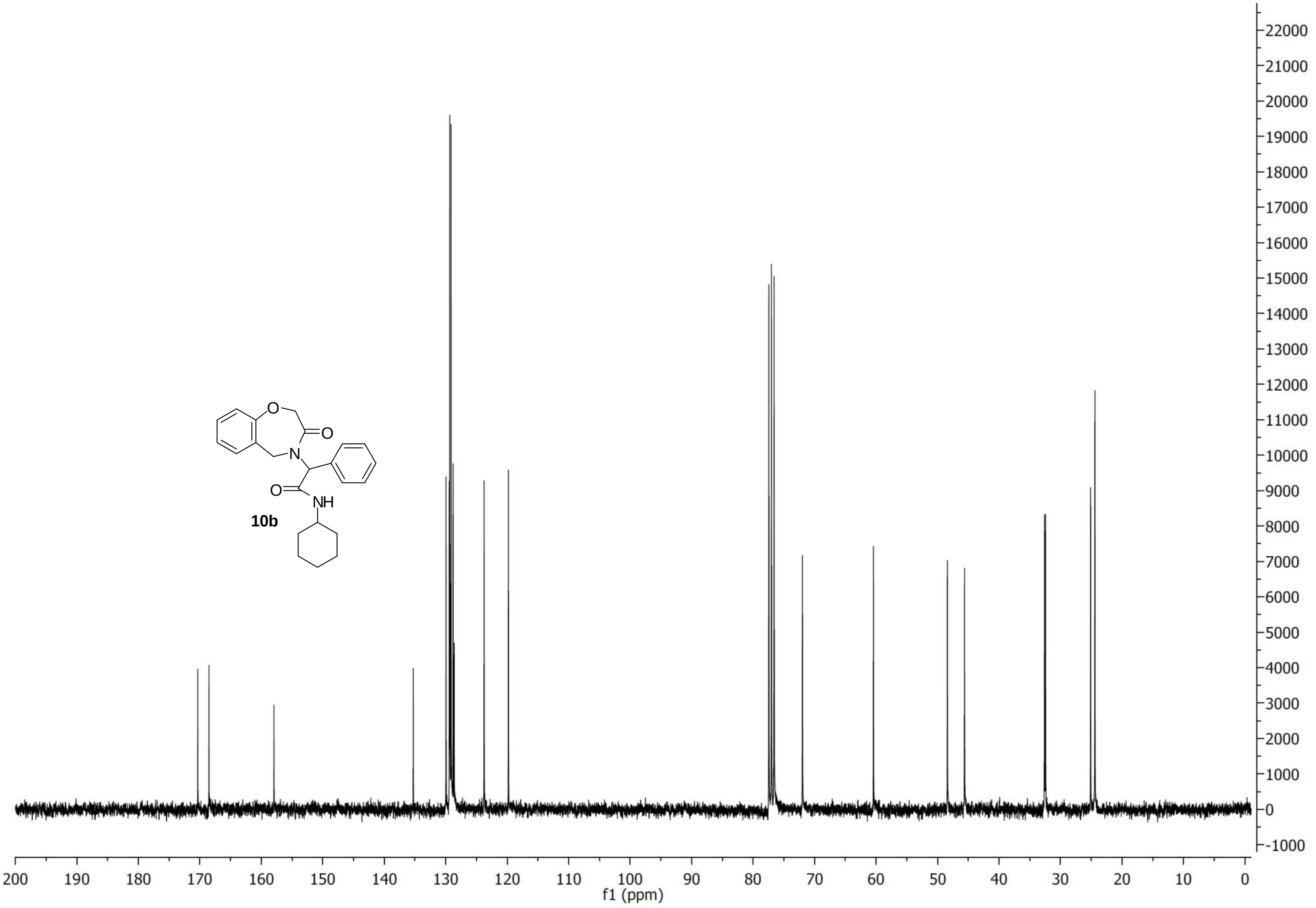
10b
O
O
N
O
NH
f1 (ppm)
200 190 180 170 160 150 140 130 120 110 100 90 80 70 60 50 40 30 20 10 0
22000 21000 20000 19000 18000 17000 16000 15000 14000 13000 12000 11000 10000 9000 8000 7000 6000 5000 4000 3000 2000 1000 0 -1000

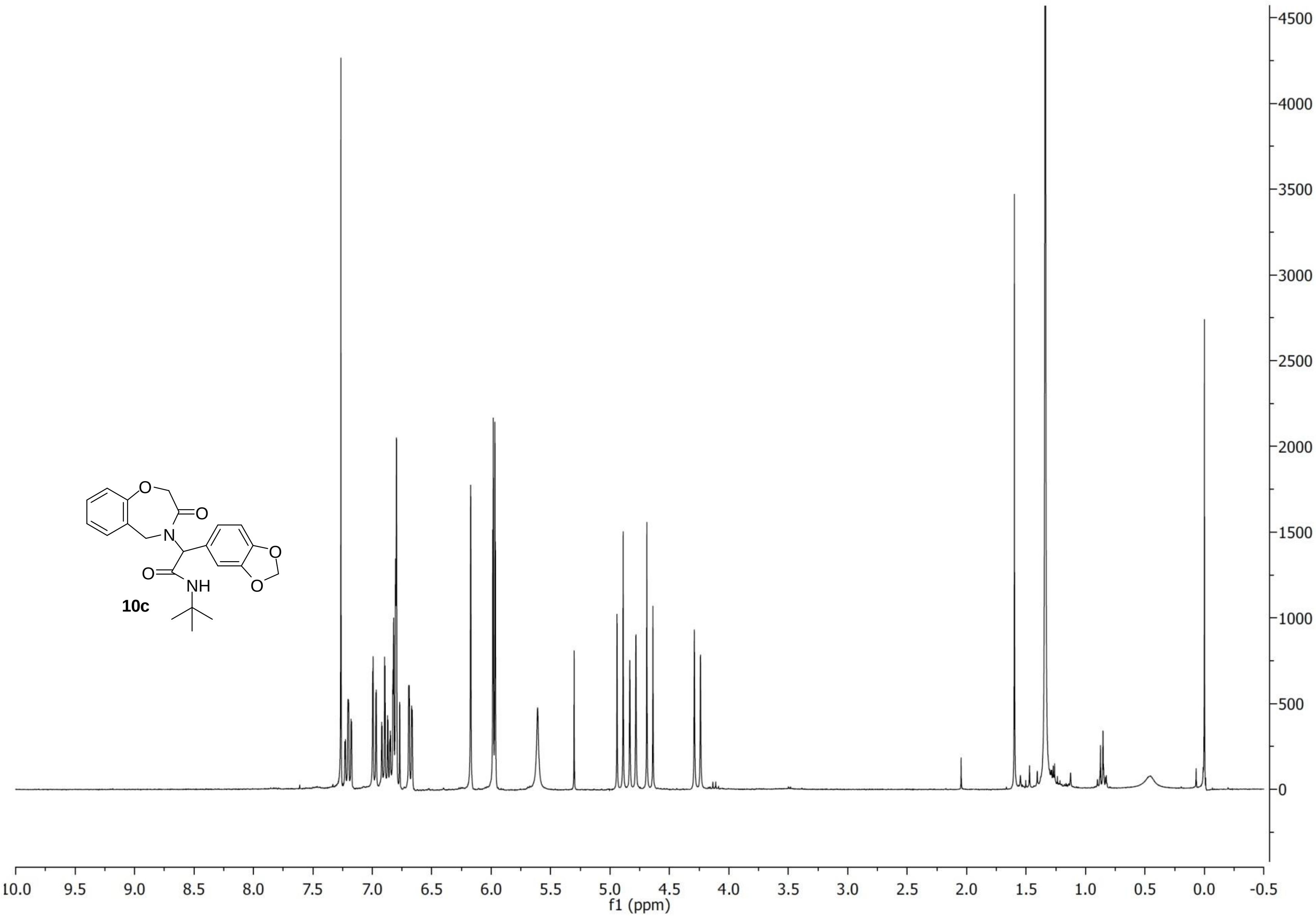
10c
O
N
NH
f1 (ppm)
10.0
9.5
9.0
8.5
8.0
7.5
7.0
6.5
6.0
5.5
5.0
4.5
4.0
3.5
3.0
2.5
2.0
1.5
1.0
0.5
0.0
-0.5
4500
4000
3500
3000
2500
2000
1500
1000
500
0

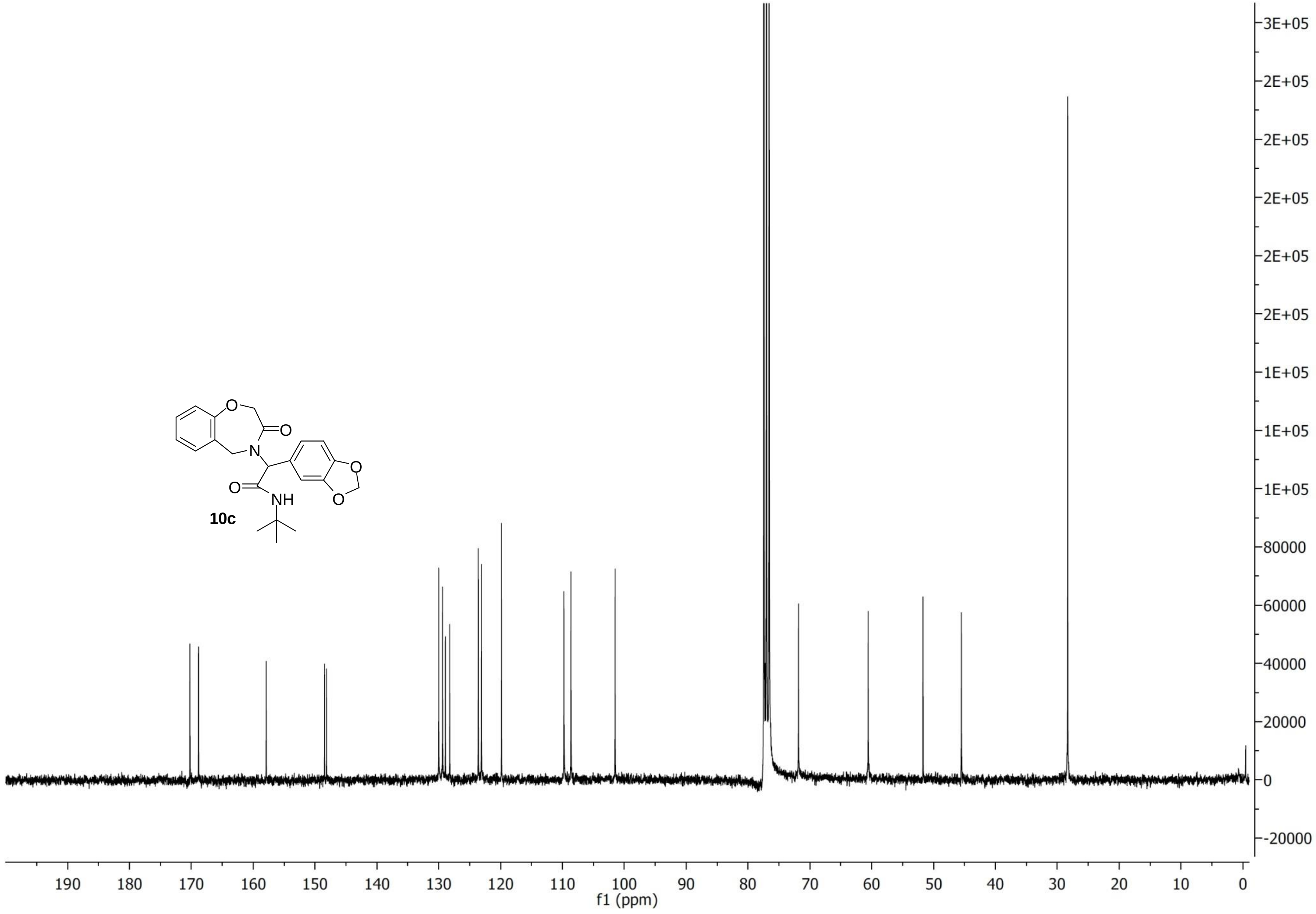
10c
O
O
N
O
O
O
NH
3E+05
2E+05
2E+05
2E+05
2E+05
2E+05
1E+05
1E+05
1E+05
80000
60000
40000
20000
0
-20000
190 180 170 160 150 140 130 120 110 100 90 80 70 60 50 40 30 20 10 0
f1 (ppm)

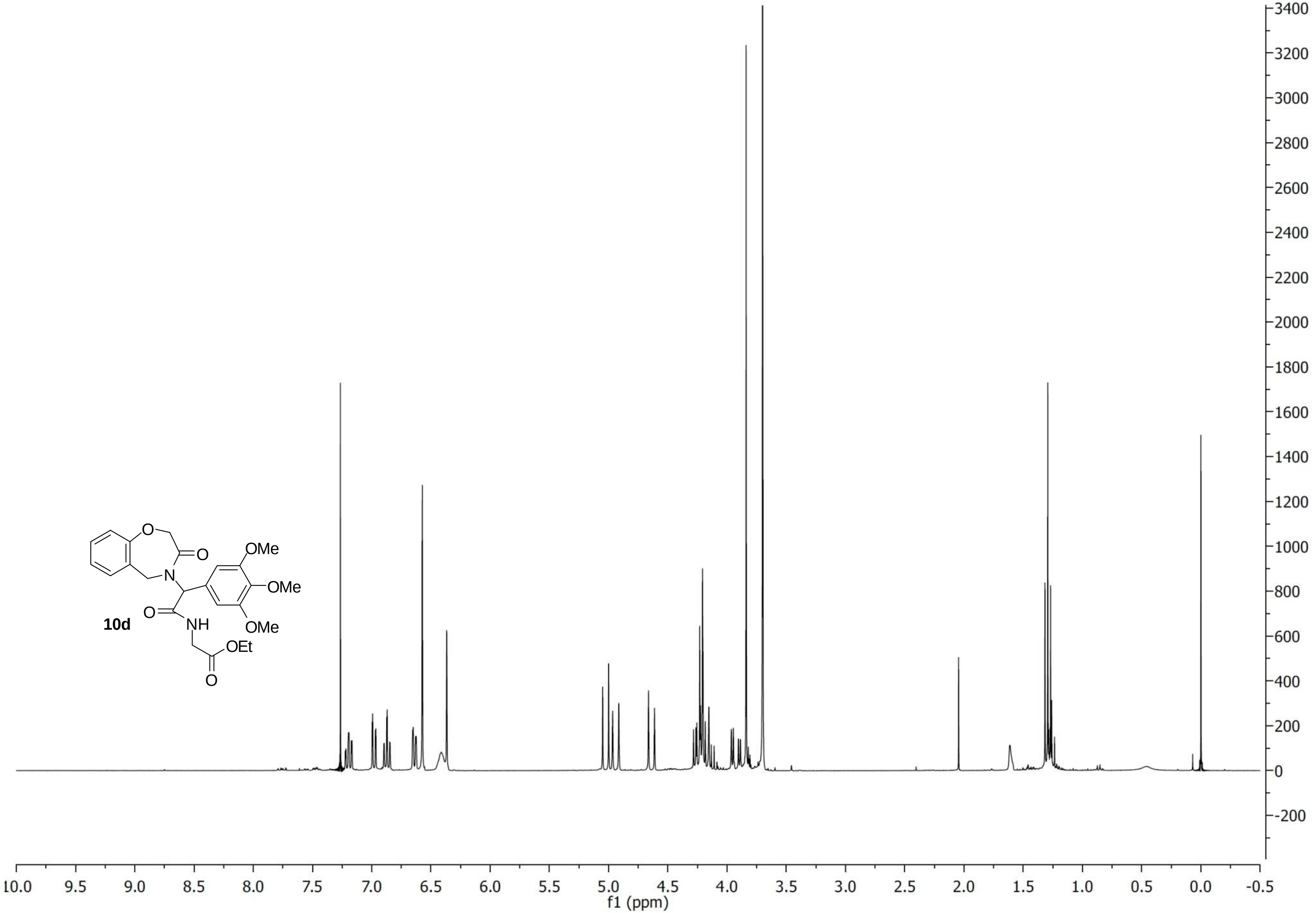
OMe
OMe
OMe
O
O
N
NH
OEt
O
10d
3400
3200
3000
2800
2600
2400
2200
2000
1800
1600
1400
1200
1000
800
600
400
200
0
-200
10.0 9.5 9.0 8.5 8.0 7.5 7.0 6.5 6.0 5.5 5.0 4.5 4.0 3.5 3.0 2.5 2.0 1.5 1.0 0.5 0.0 -0.5
f1 (ppm)

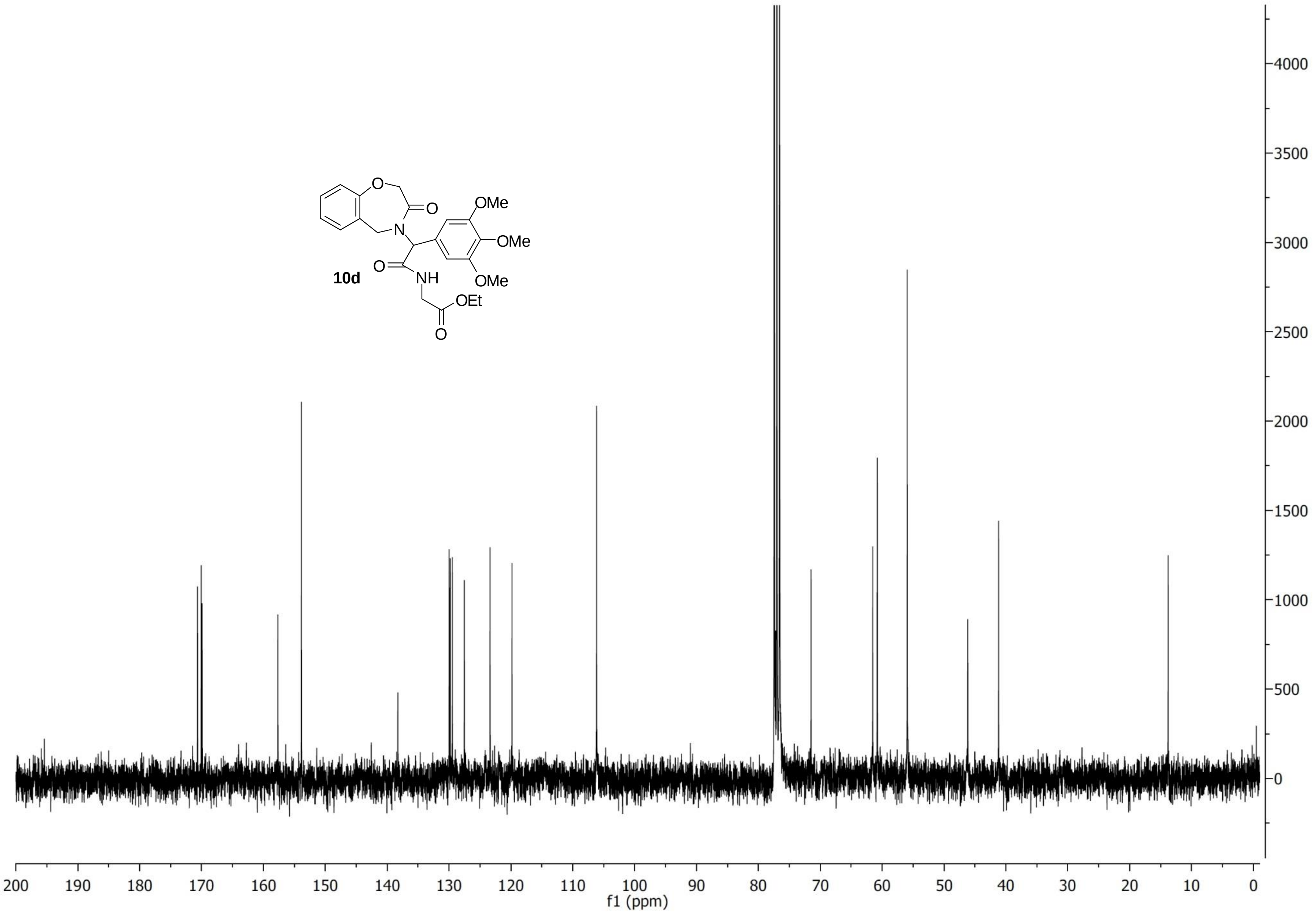
10d
OMe
OMe
OMe
NH
OEt
f1 (ppm)

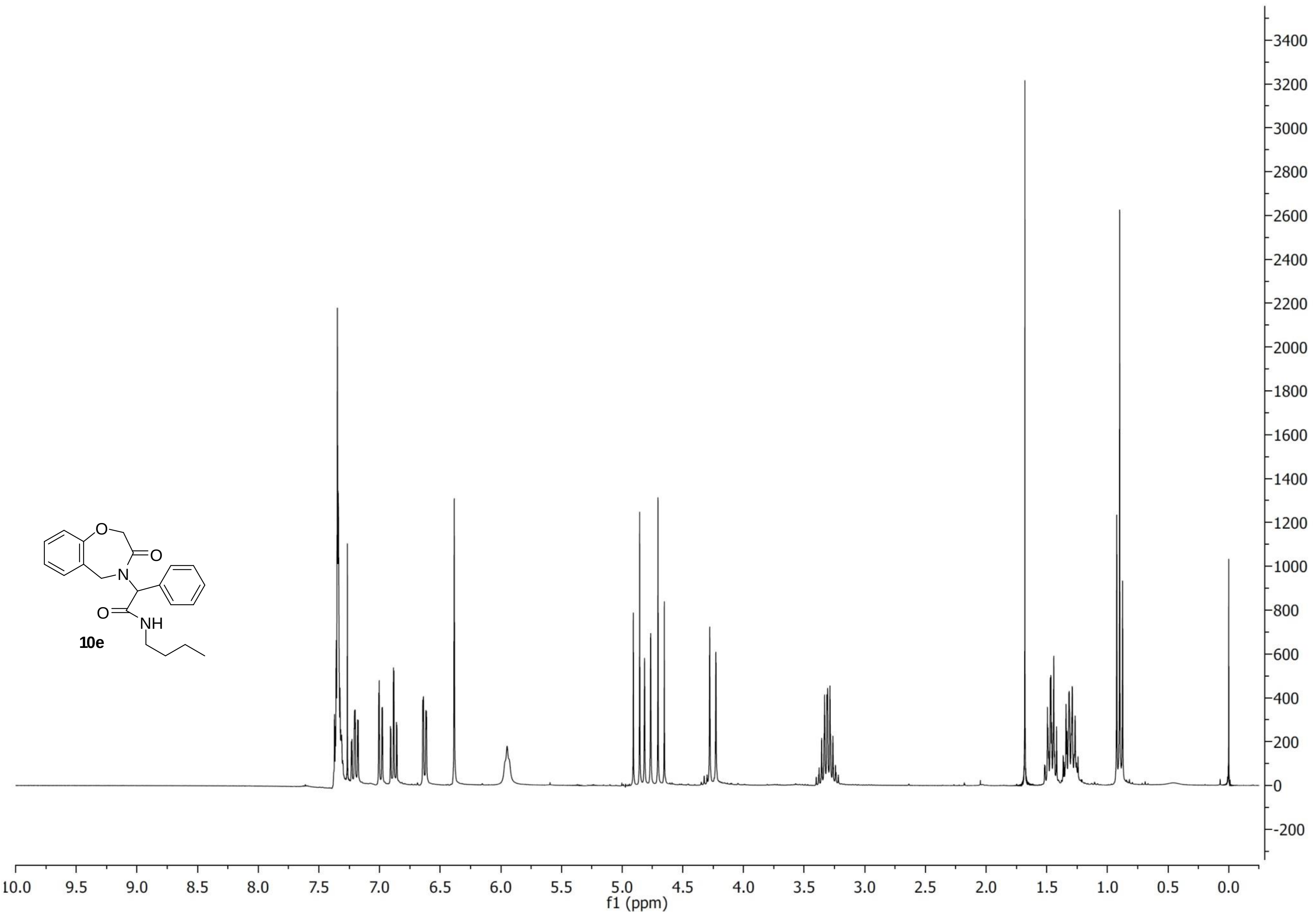
O
O
N
O
NH
10e
10.0 9.5 9.0 8.5 8.0 7.5 7.0 6.5 6.0 5.5 5.0 4.5 4.0 3.5 3.0 2.5 2.0 1.5 1.0 0.5 0.0
f1 (ppm)
3400 3200 3000 2800 2600 2400 2200 2000 1800 1600 1400 1200 1000 800 600 400 200 0 -200

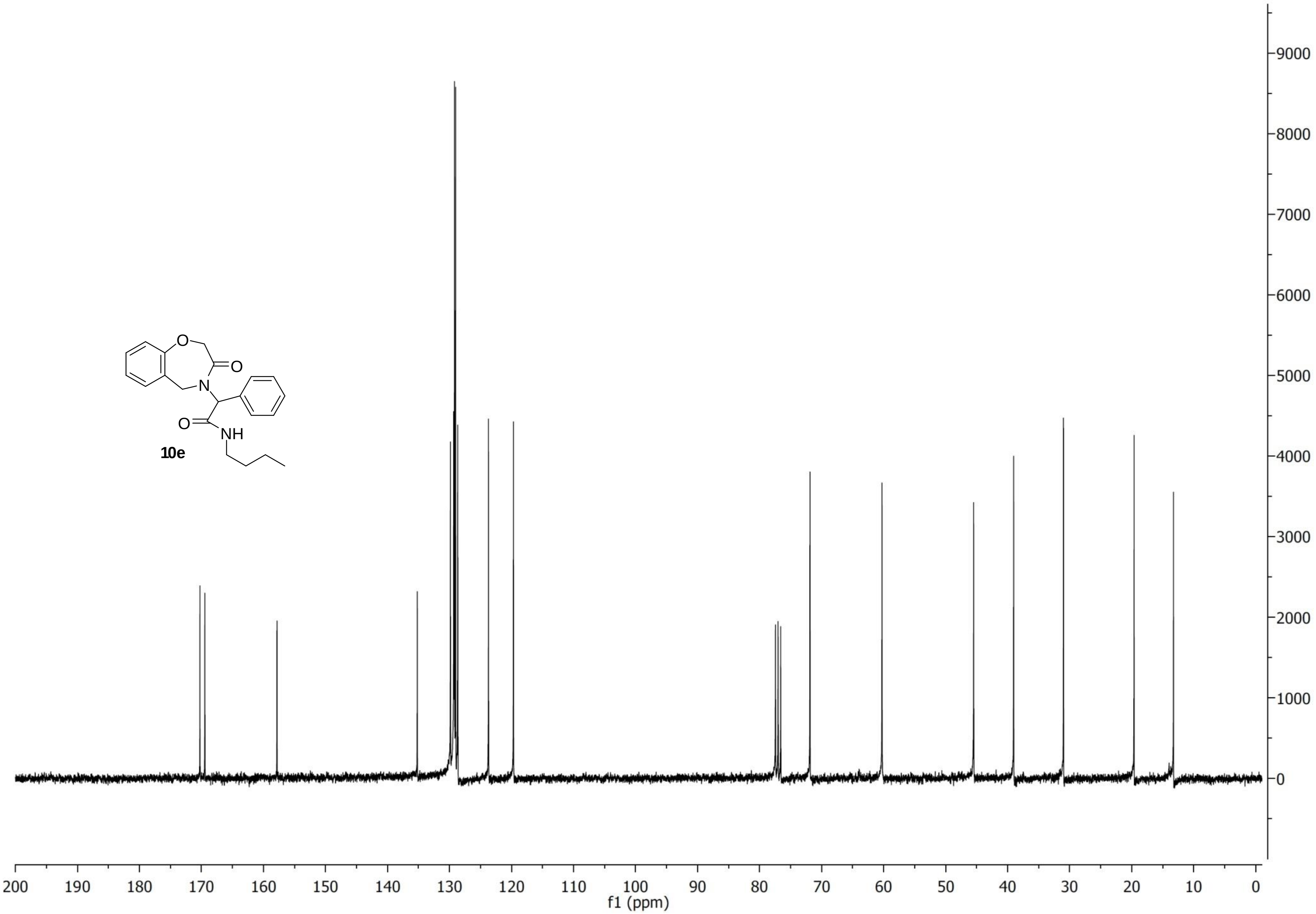
O
O
N
O
NH
10e
9000
8000
7000
6000
5000
4000
3000
2000
1000
0
200 190 180 170 160 150 140 130 120 110 100 90 80 70 60 50 40 30 20 10 0
f1 (ppm)

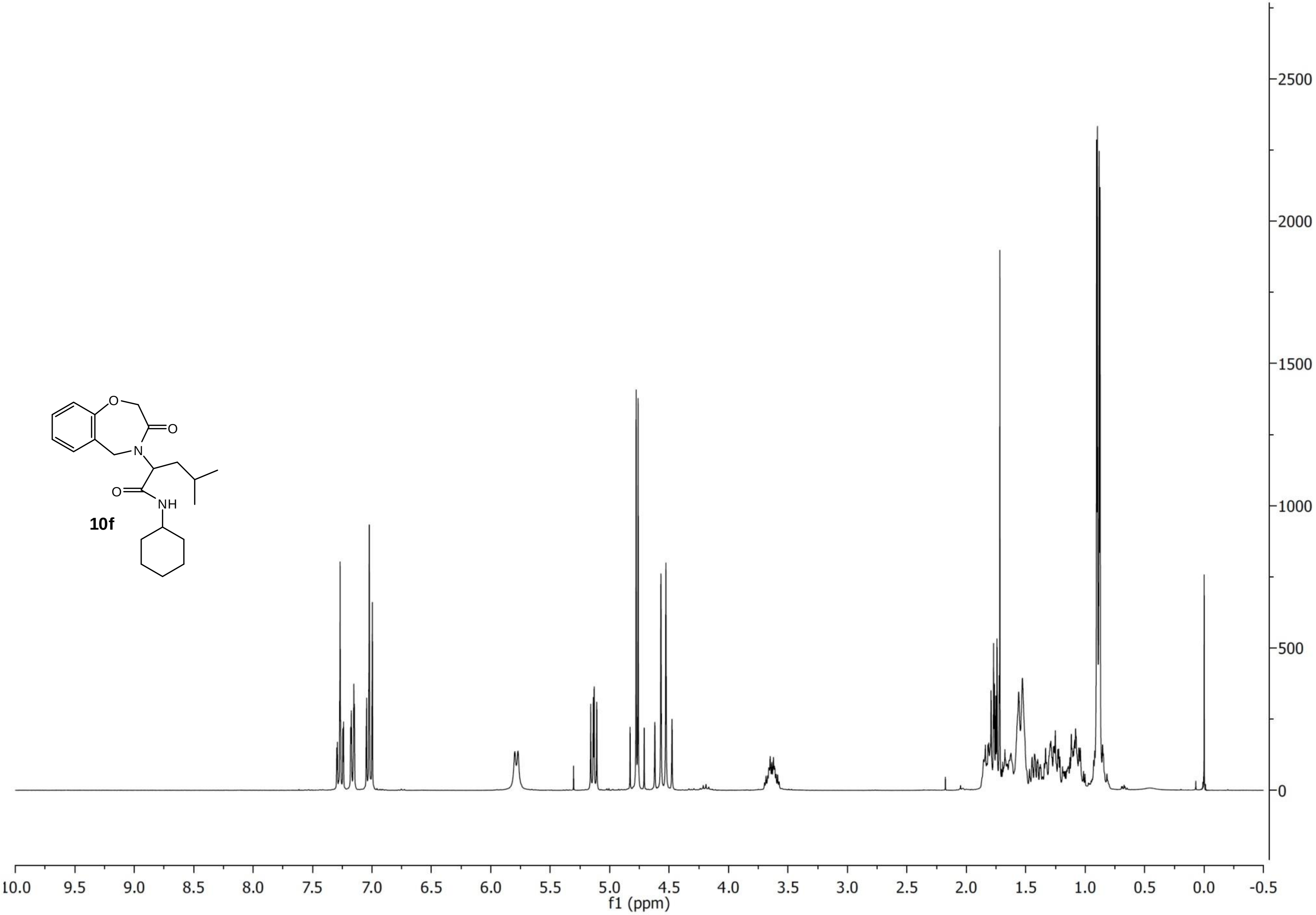
10f
O
N
NH
2500
2000
1500
1000
500
0
10.0 9.5 9.0 8.5 8.0 7.5 7.0 6.5 6.0 5.5 5.0 4.5 4.0 3.5 3.0 2.5 2.0 1.5 1.0 0.5 0.0 -0.5
f1 (ppm)

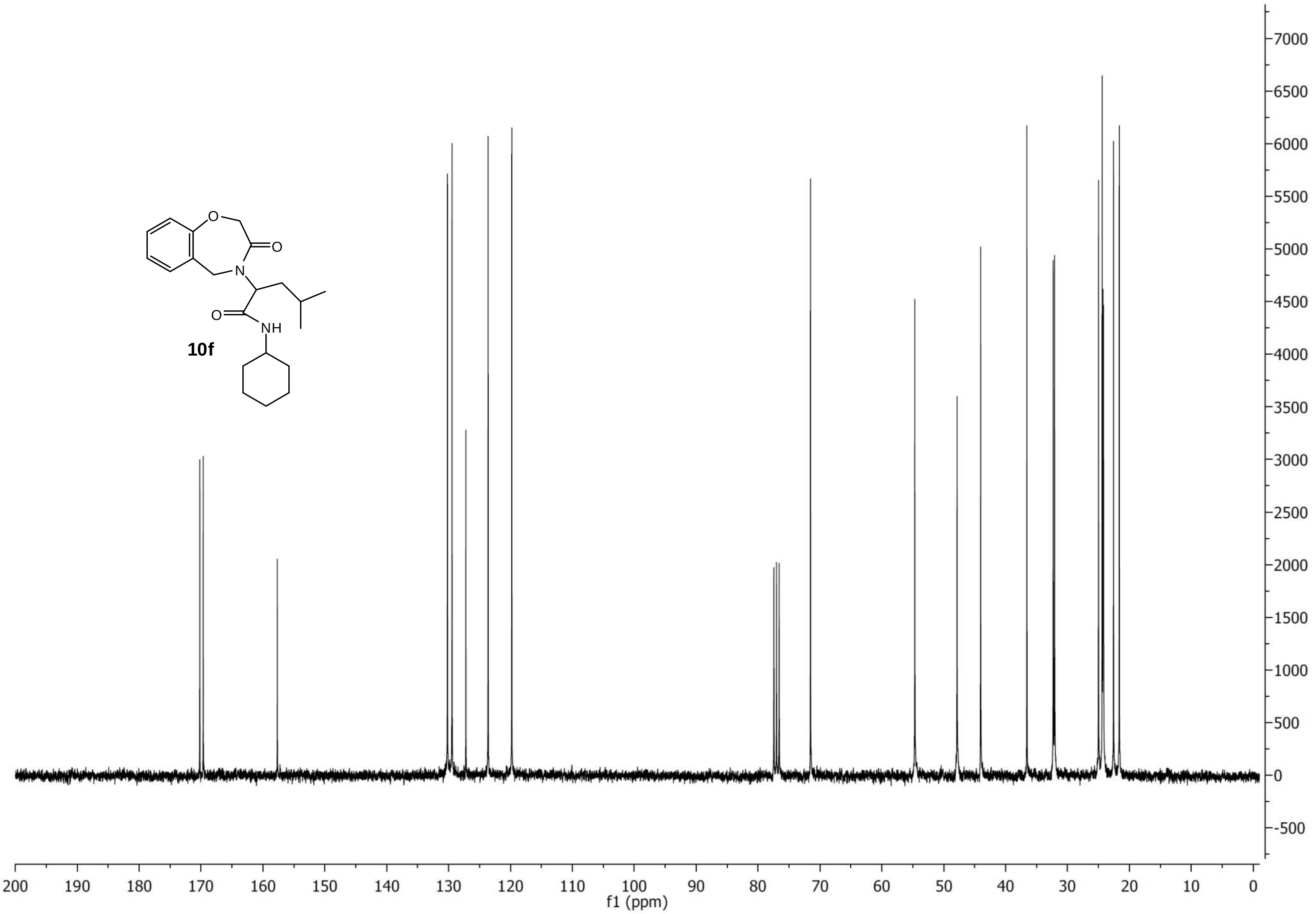

10f
O
NH
N
f1 (ppm)

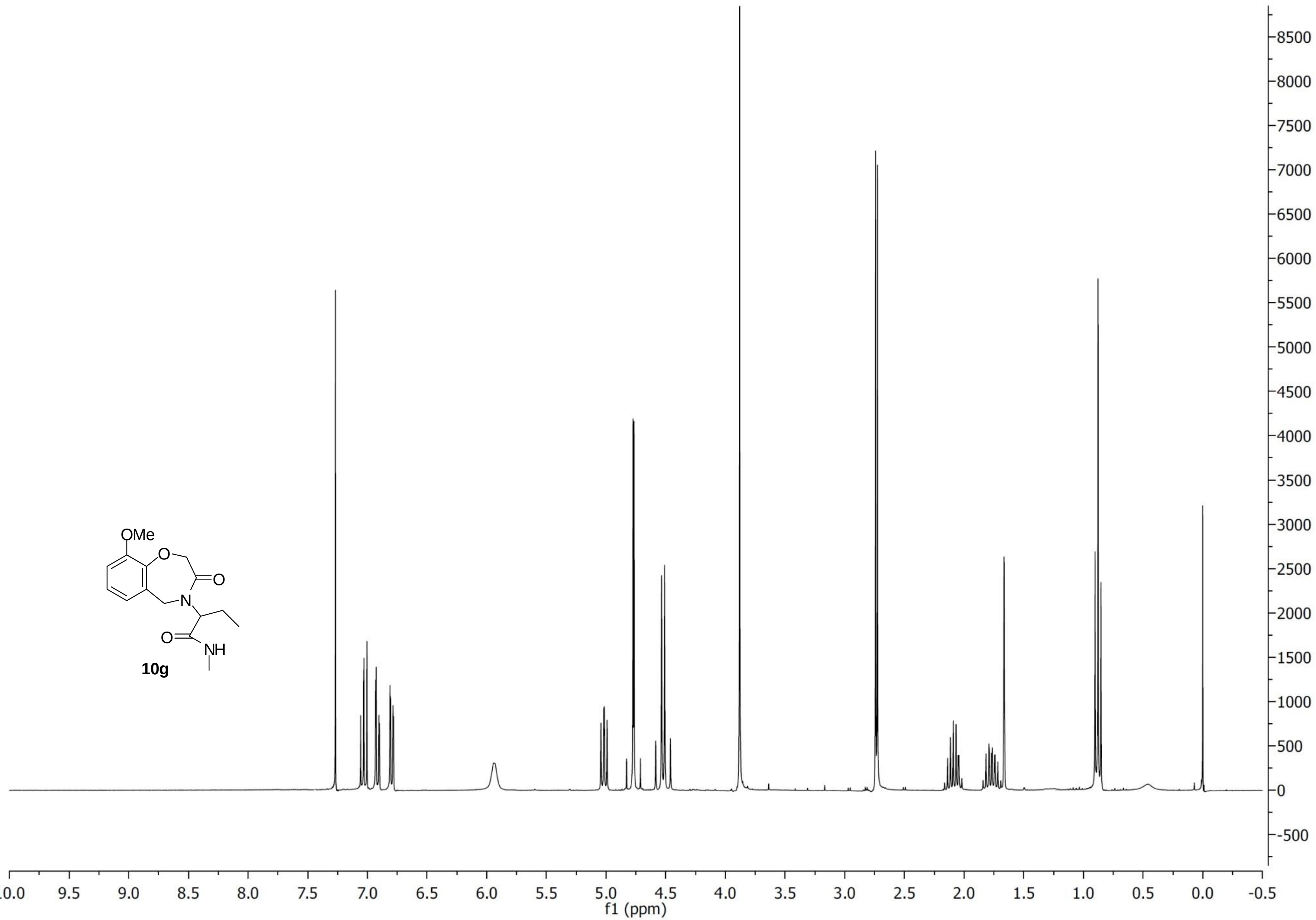
OMe
O
O
N
O
NH
10g
8500
8000
7500
7000
6500
6000
5500
5000
4500
4000
3500
3000
2500
2000
1500
1000
500
0
-500
10.0 9.5 9.0 8.5 8.0 7.5 7.0 6.5 6.0 5.5 5.0 4.5 4.0 3.5 3.0 2.5 2.0 1.5 1.0 0.5 0.0 -0.5
f1 (ppm)

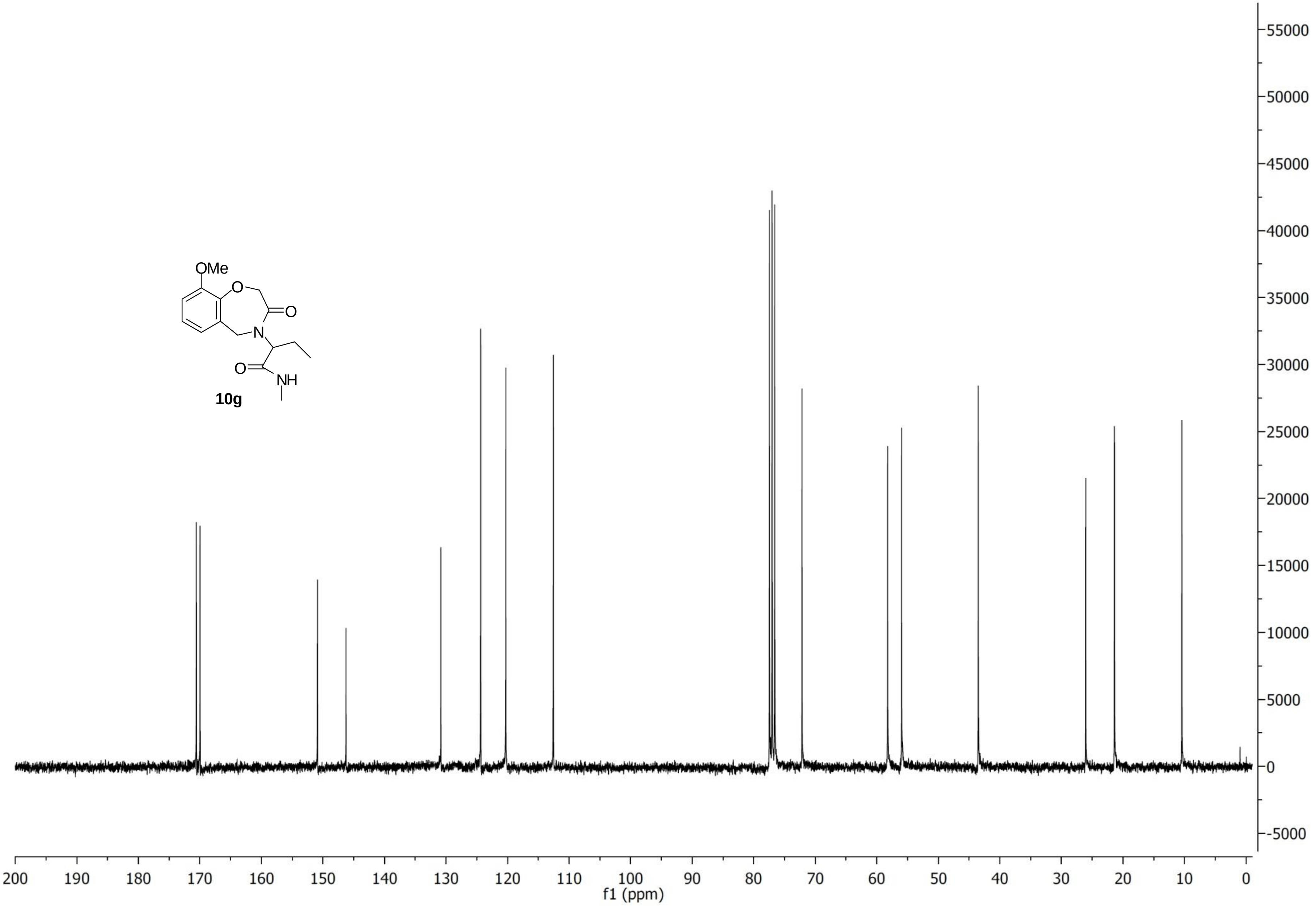

OMe
O
O
N
O
NH
10g
55000
50000
45000
40000
35000
30000
25000
20000
15000
10000
5000
0
-5000
200 190 180 170 160 150 140 130 120 110 100 90 80 70 60 50 40 30 20 10 0
f1 (ppm)

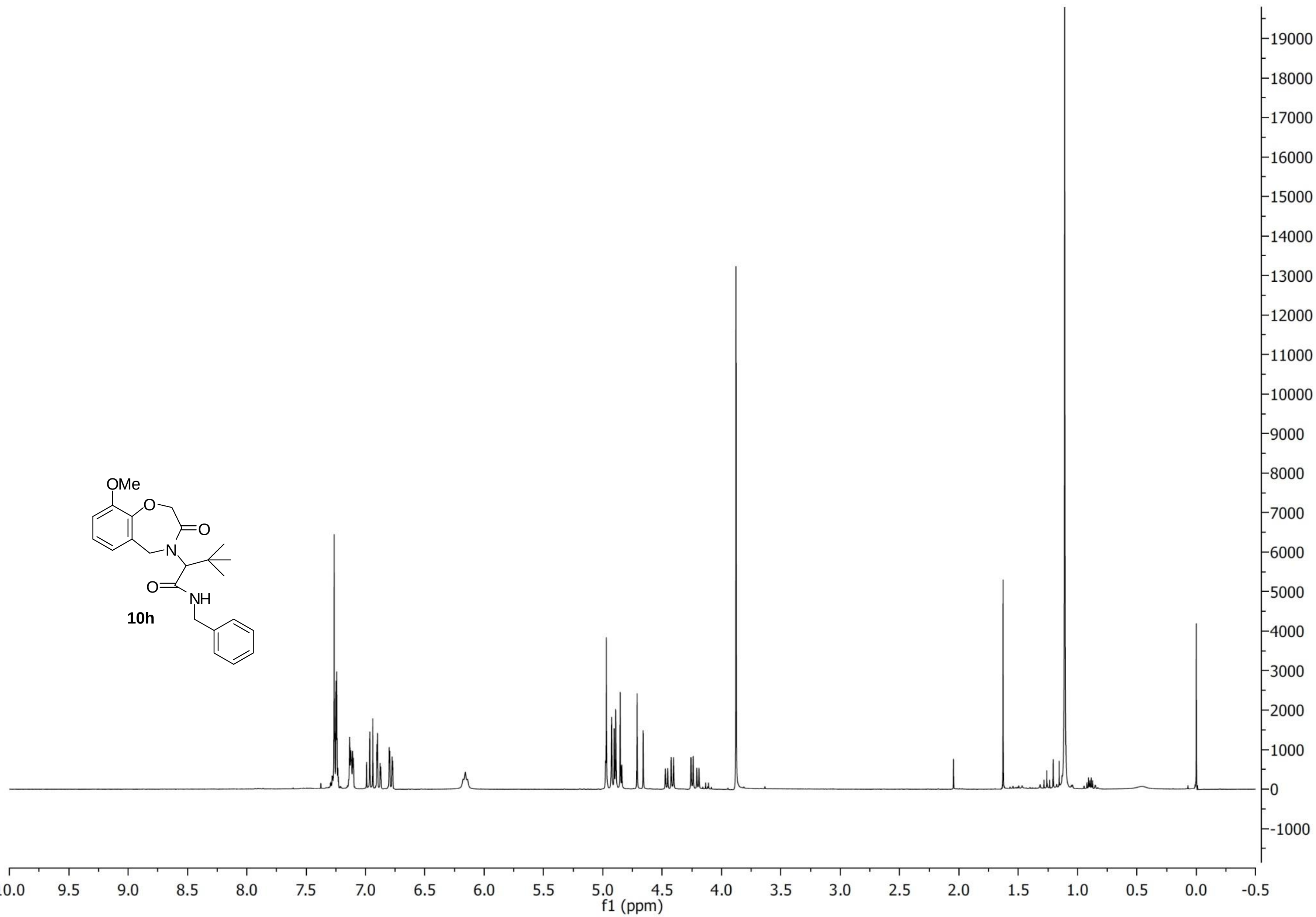
OMe
O
O
N
O
NH
10h
f1 (ppm)

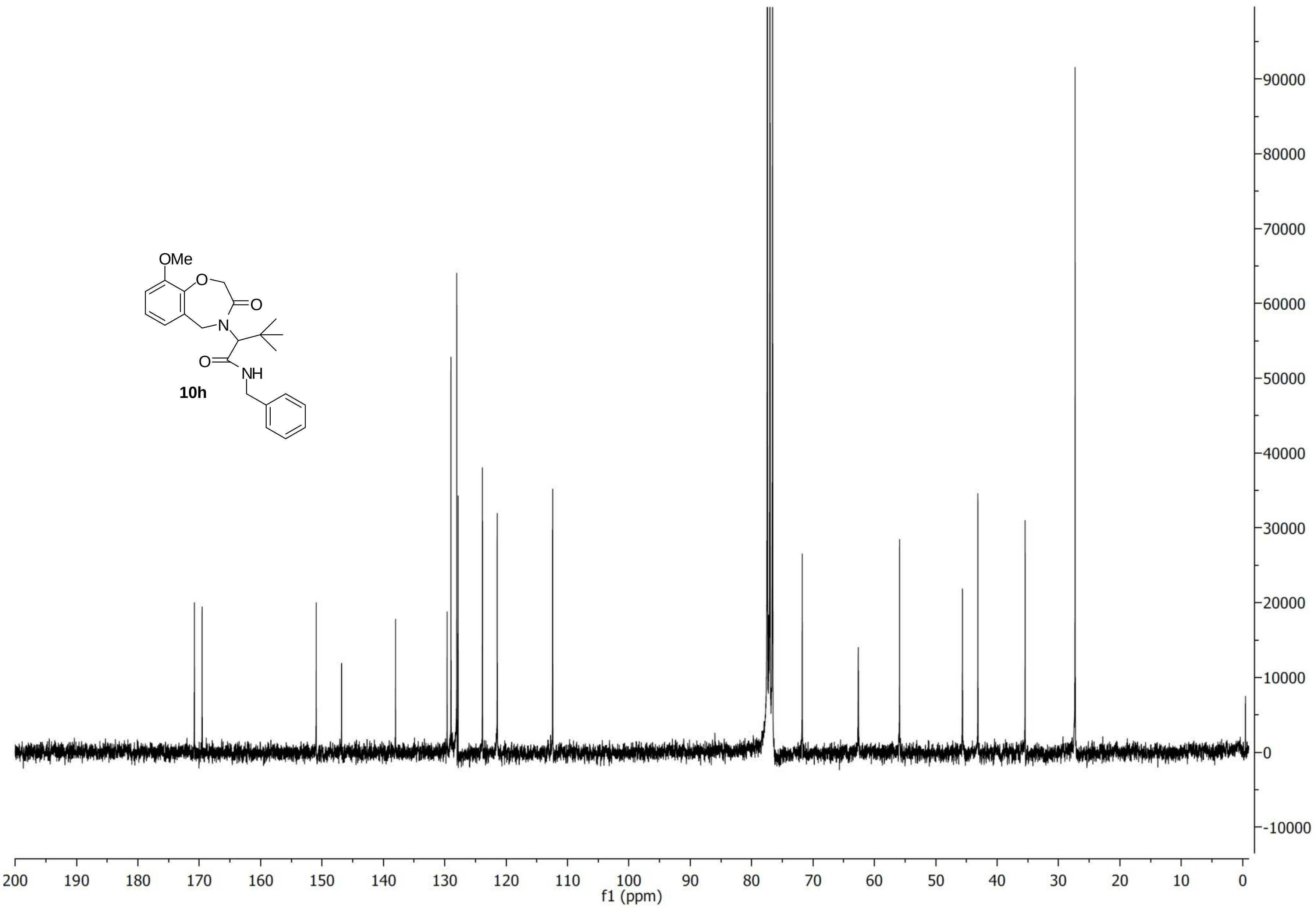
OMe
O
O
N
O
NH
10h
f1 (ppm)
200 190 180 170 160 150 140 130 120 110 100 90 80 70 60 50 40 30 20 10 0
90000 80000 70000 60000 50000 40000 30000 20000 10000 0 -10000

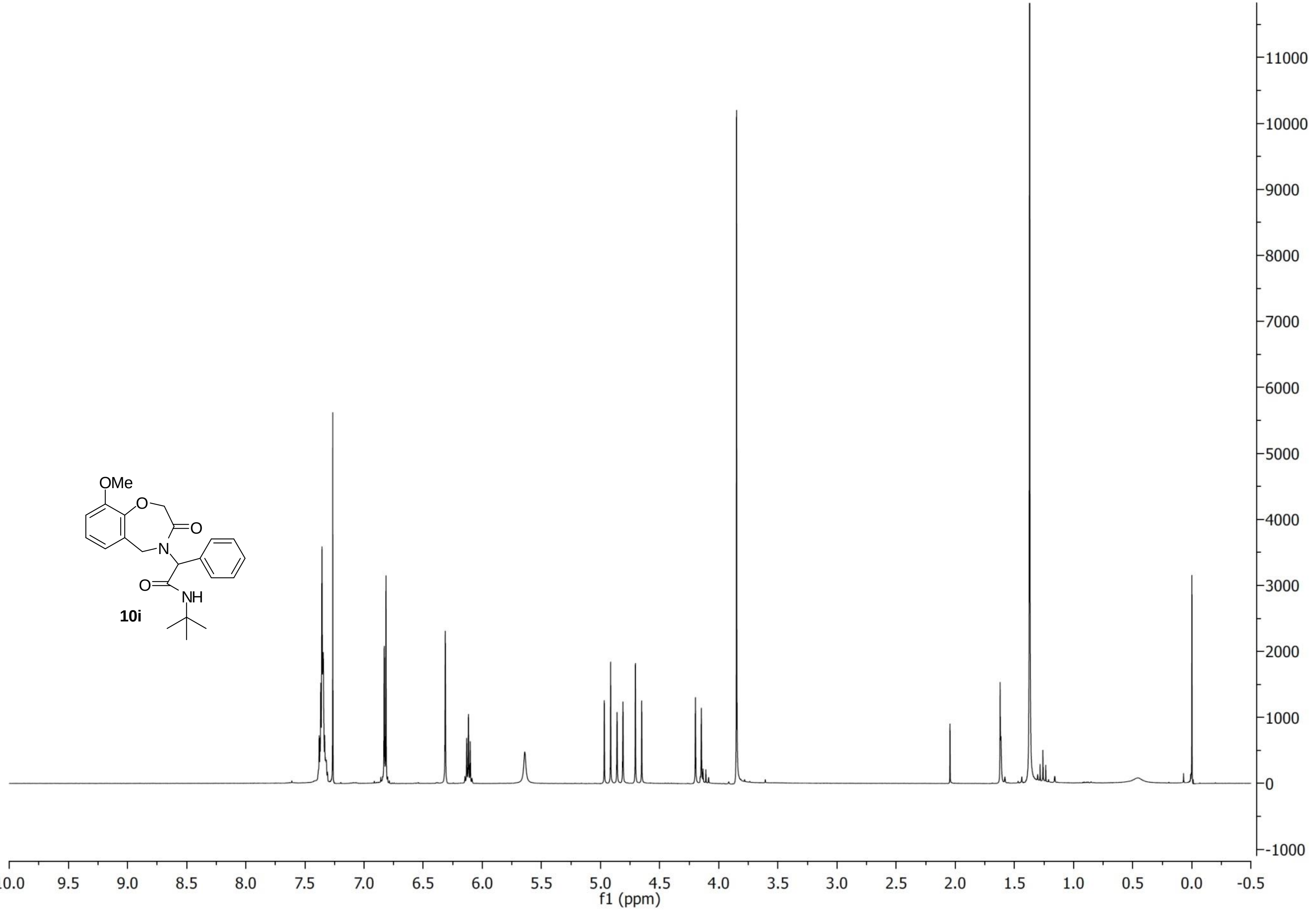
OMe
O
O
N
O
NH
10i
11000
10000
9000
8000
7000
6000
5000
4000
3000
2000
1000
0
-1000
10.0 9.5 9.0 8.5 8.0 7.5 7.0 6.5 6.0 5.5 5.0 4.5 4.0 3.5 3.0 2.5 2.0 1.5 1.0 0.5 0.0 -0.5
f1 (ppm)

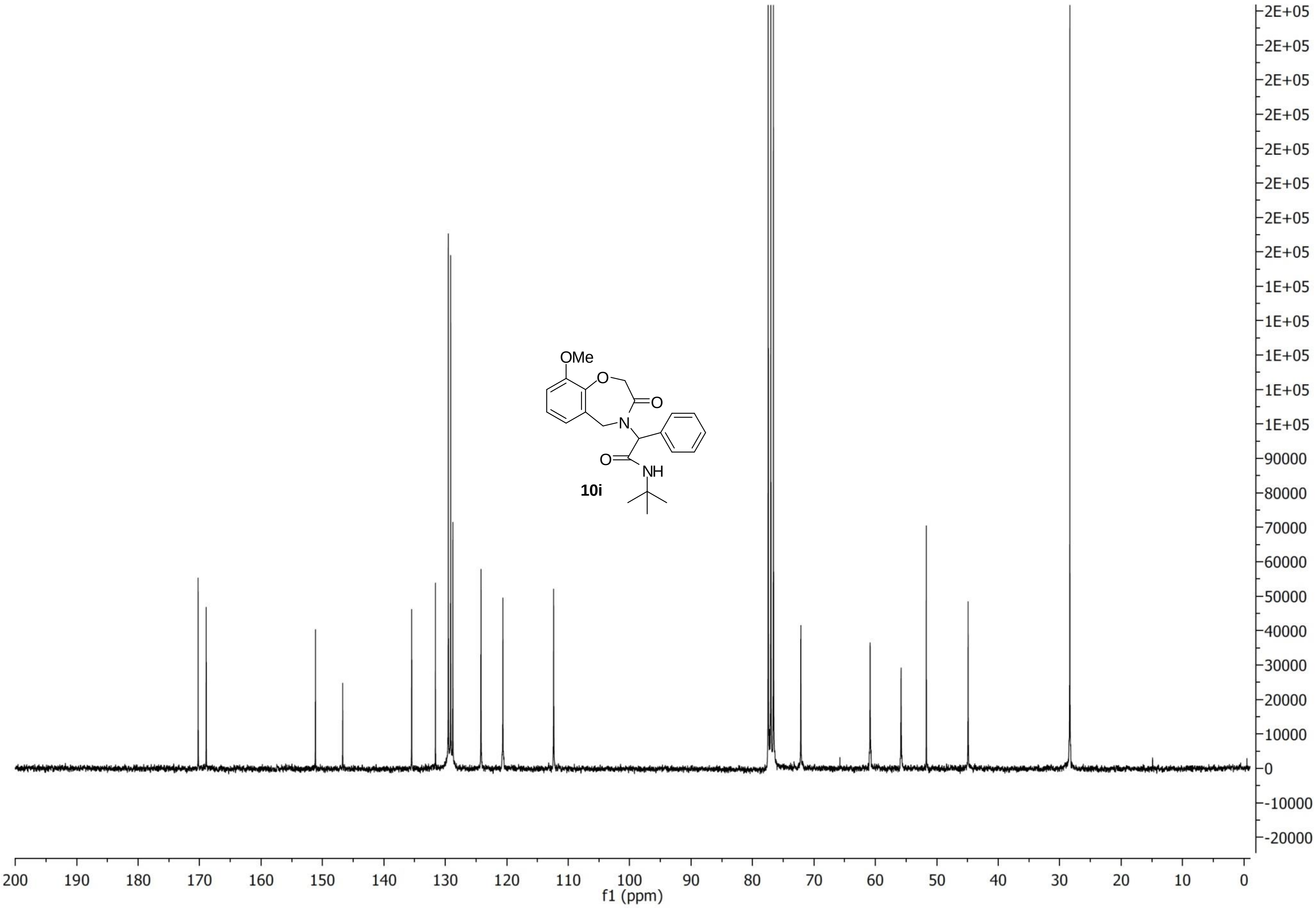
OMe
O
O
N
O
NH
10i
f1 (ppm)
200 190 180 170 160 150 140 130 120 110 100 90 80 70 60 50 40 30 20 10 0
2E+05
1E+05
90000
80000
70000
60000
50000
40000
30000
20000
10000
0
-10000
-20000

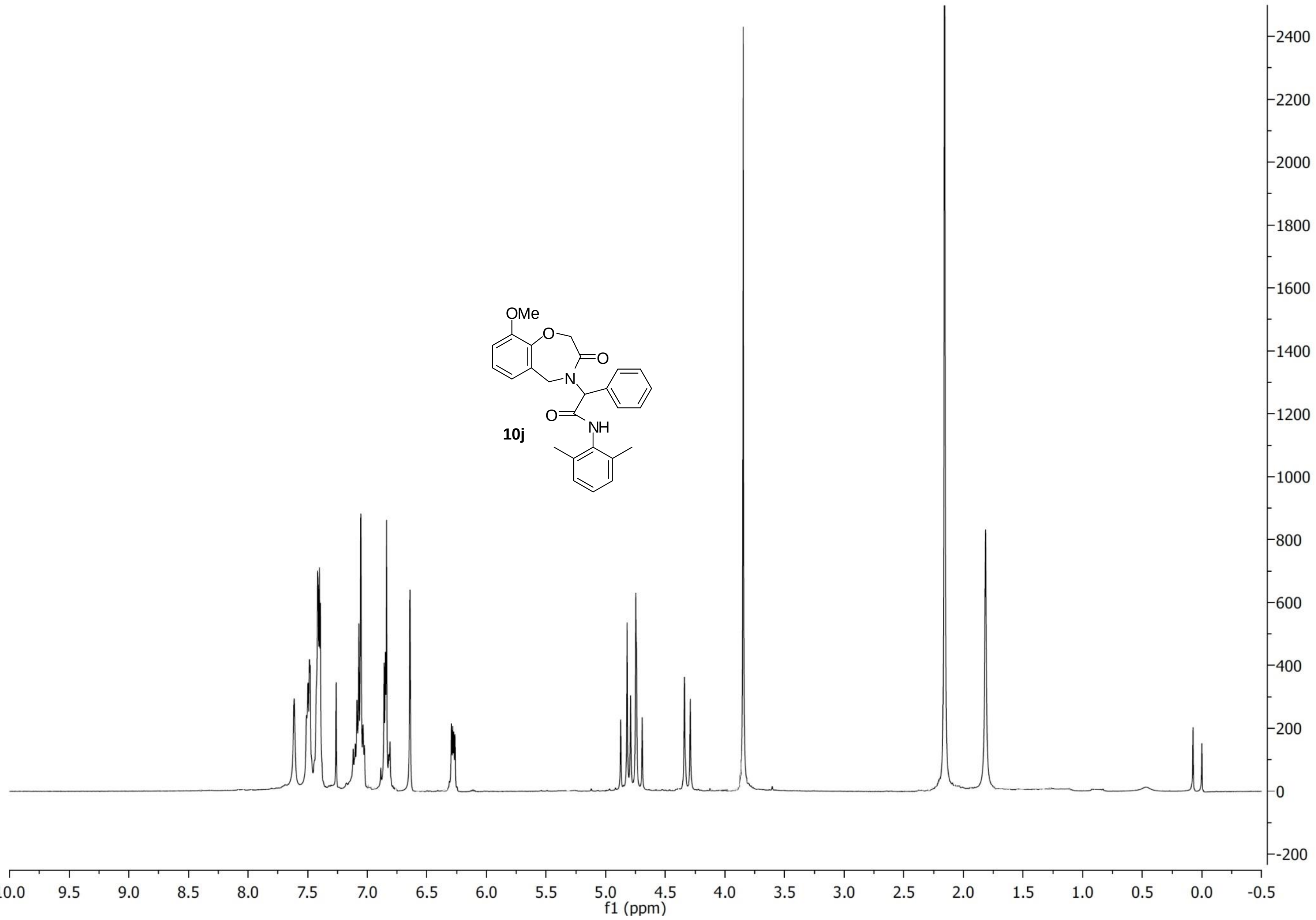
OMe
O
O
N
O
NH
10j
2400
2200
2000
1800
1600
1400
1200
1000
800
600
400
200
0
-200
10.0 9.5 9.0 8.5 8.0 7.5 7.0 6.5 6.0 5.5 5.0 4.5 4.0 3.5 3.0 2.5 2.0 1.5 1.0 0.5 0.0 -0.5
f1 (ppm)

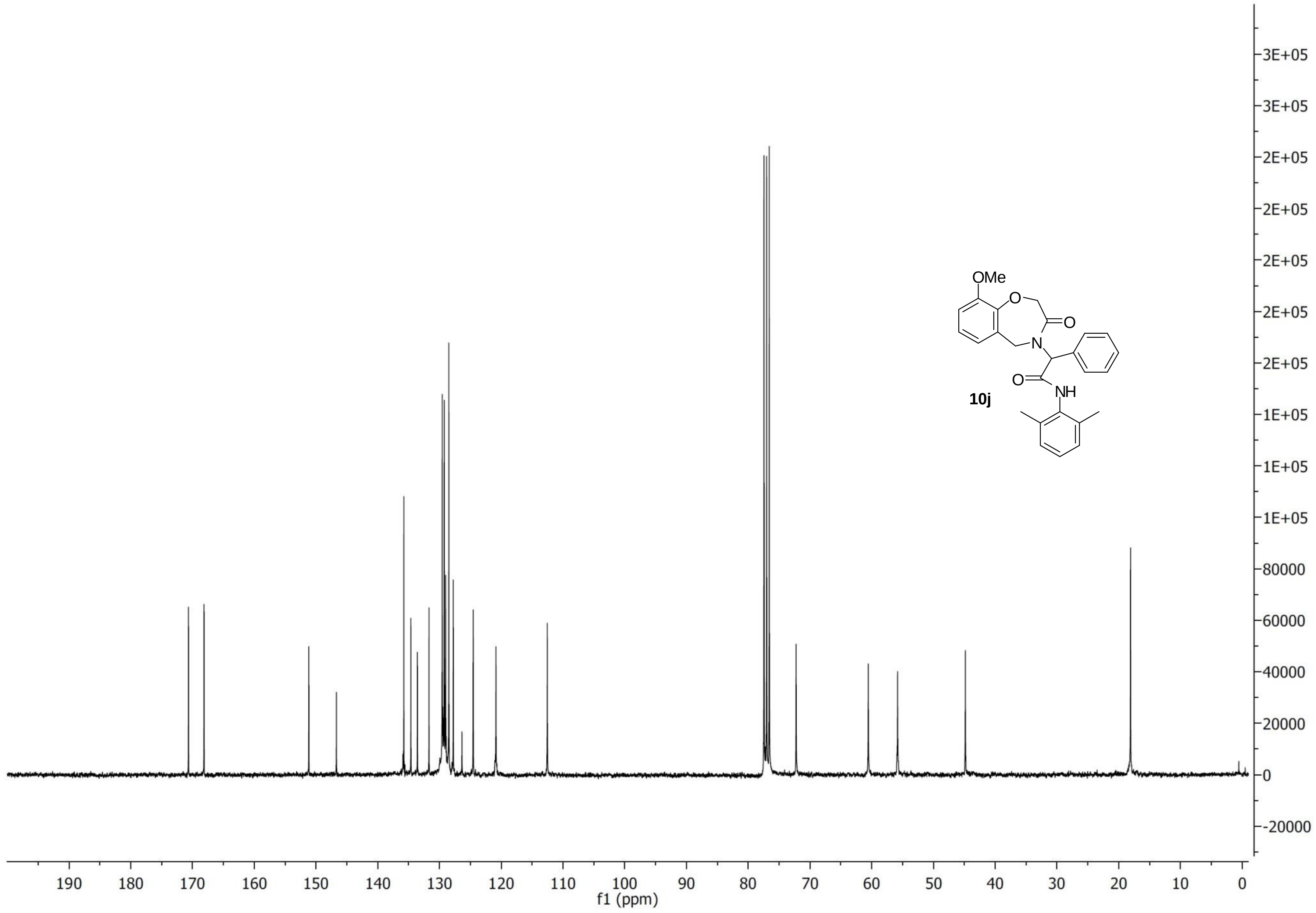
OMe
O
O
N
O
NH
10j
f1 (ppm)

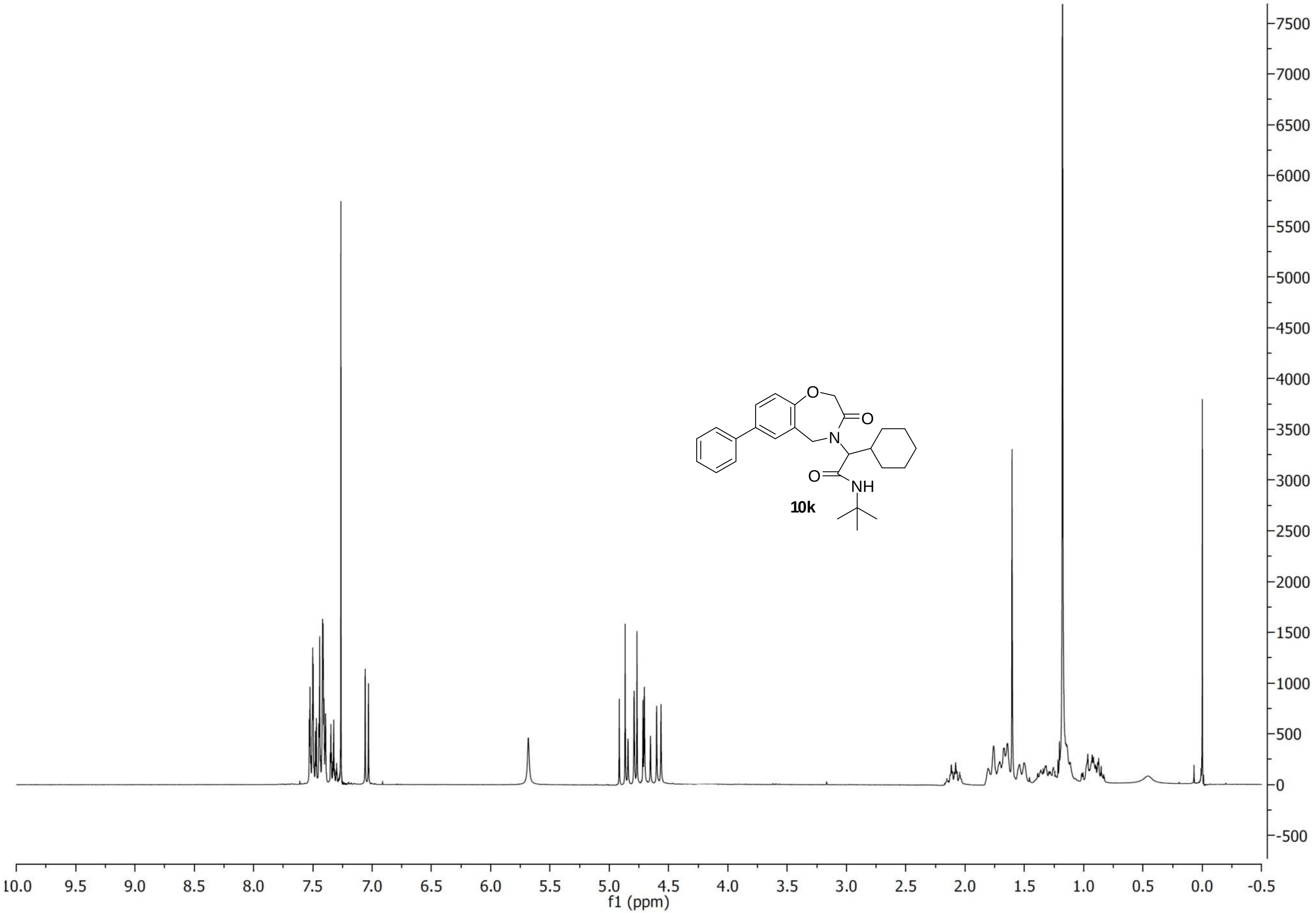
10k
f1 (ppm)

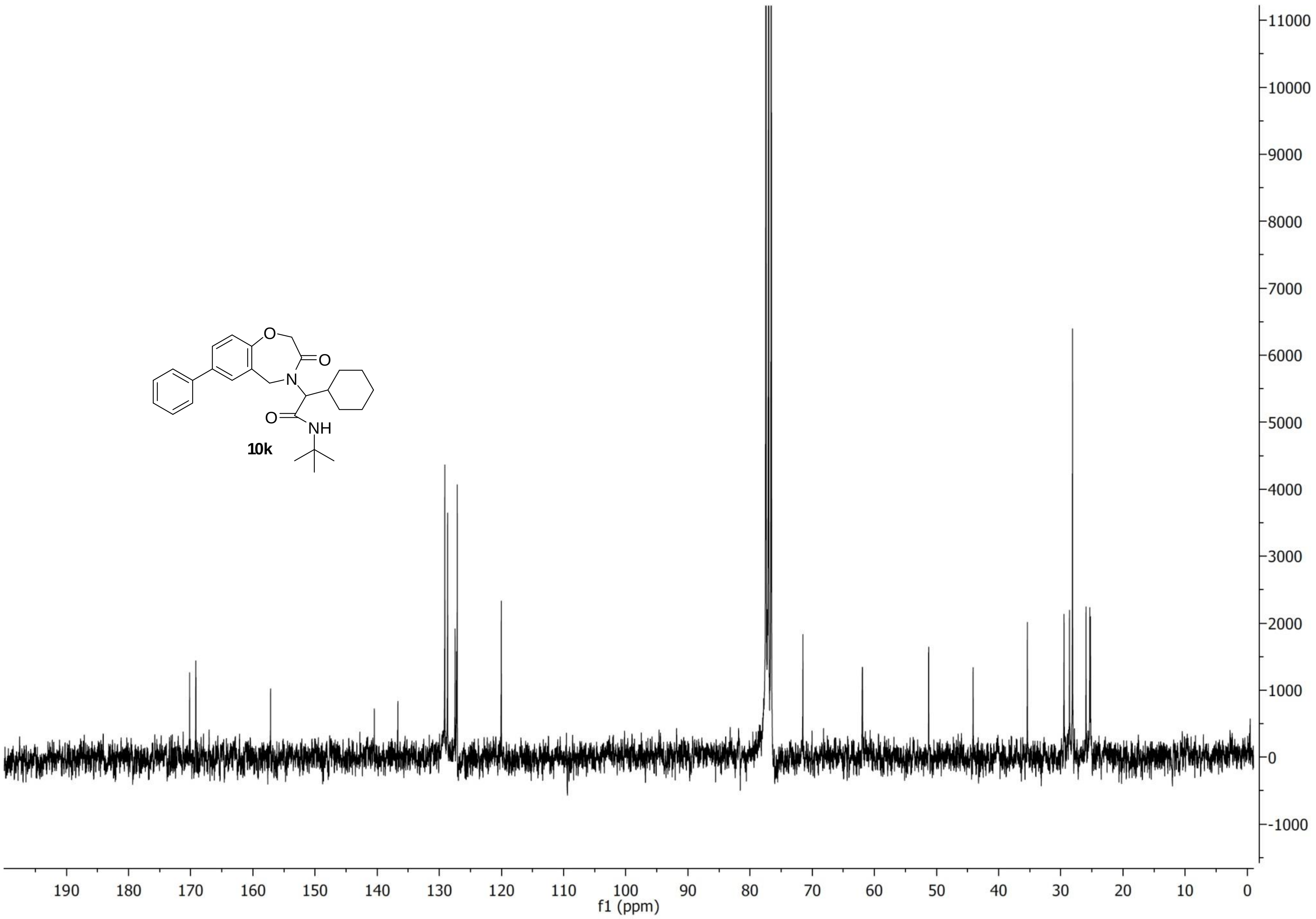
O
O
N
O
NH
10k
f1 (ppm)

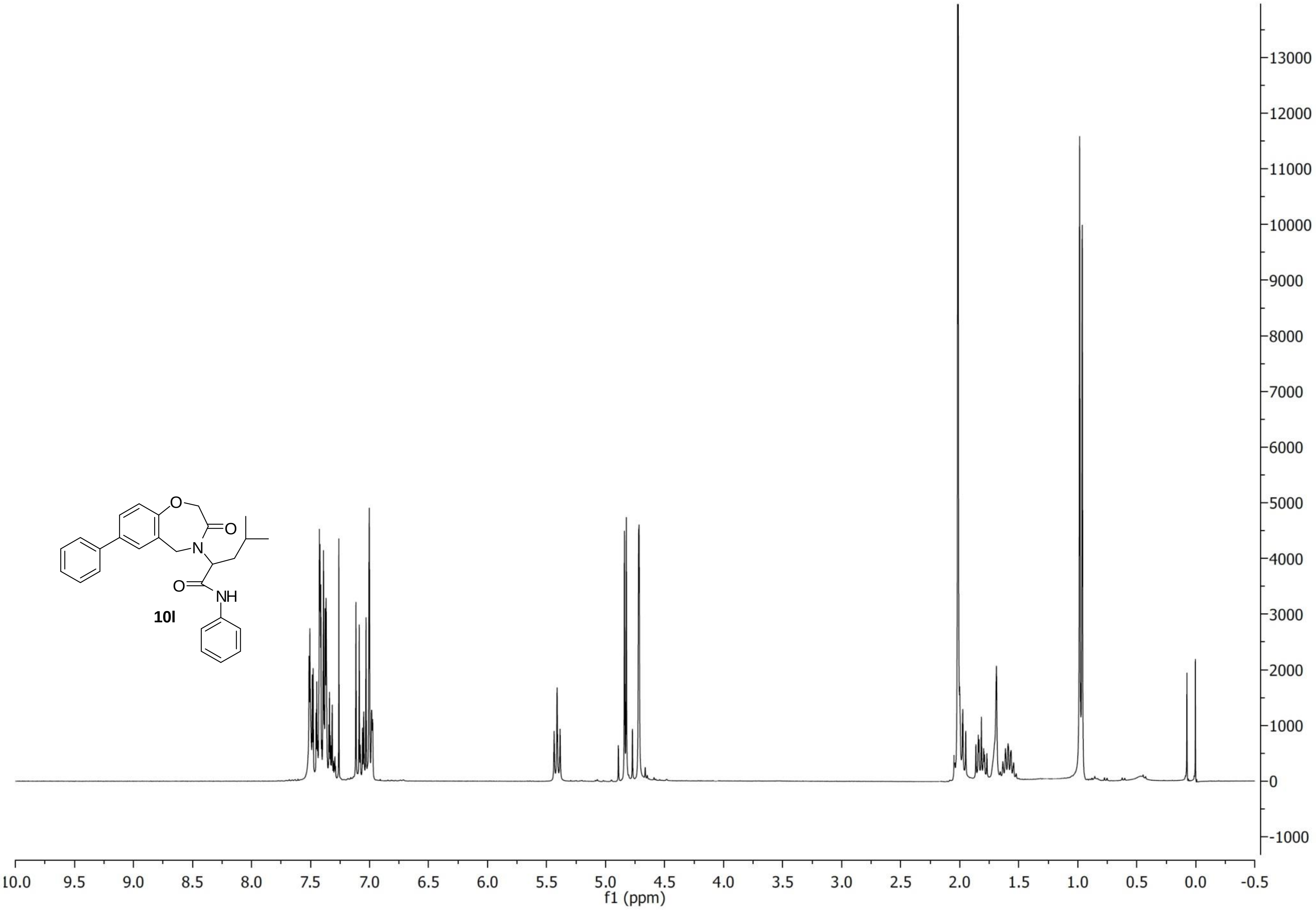
10l
f1 (ppm)

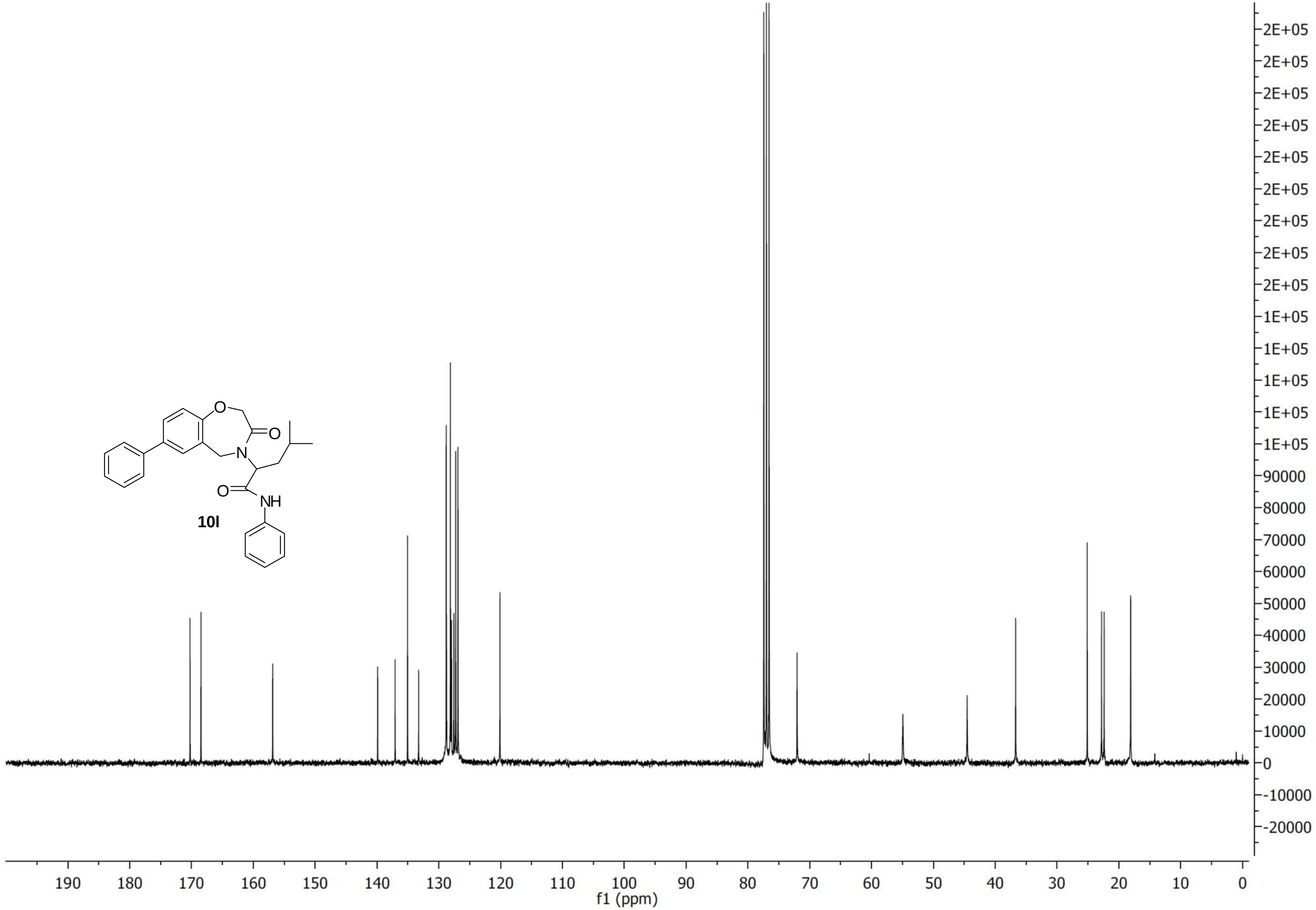
10l
f1 (ppm)

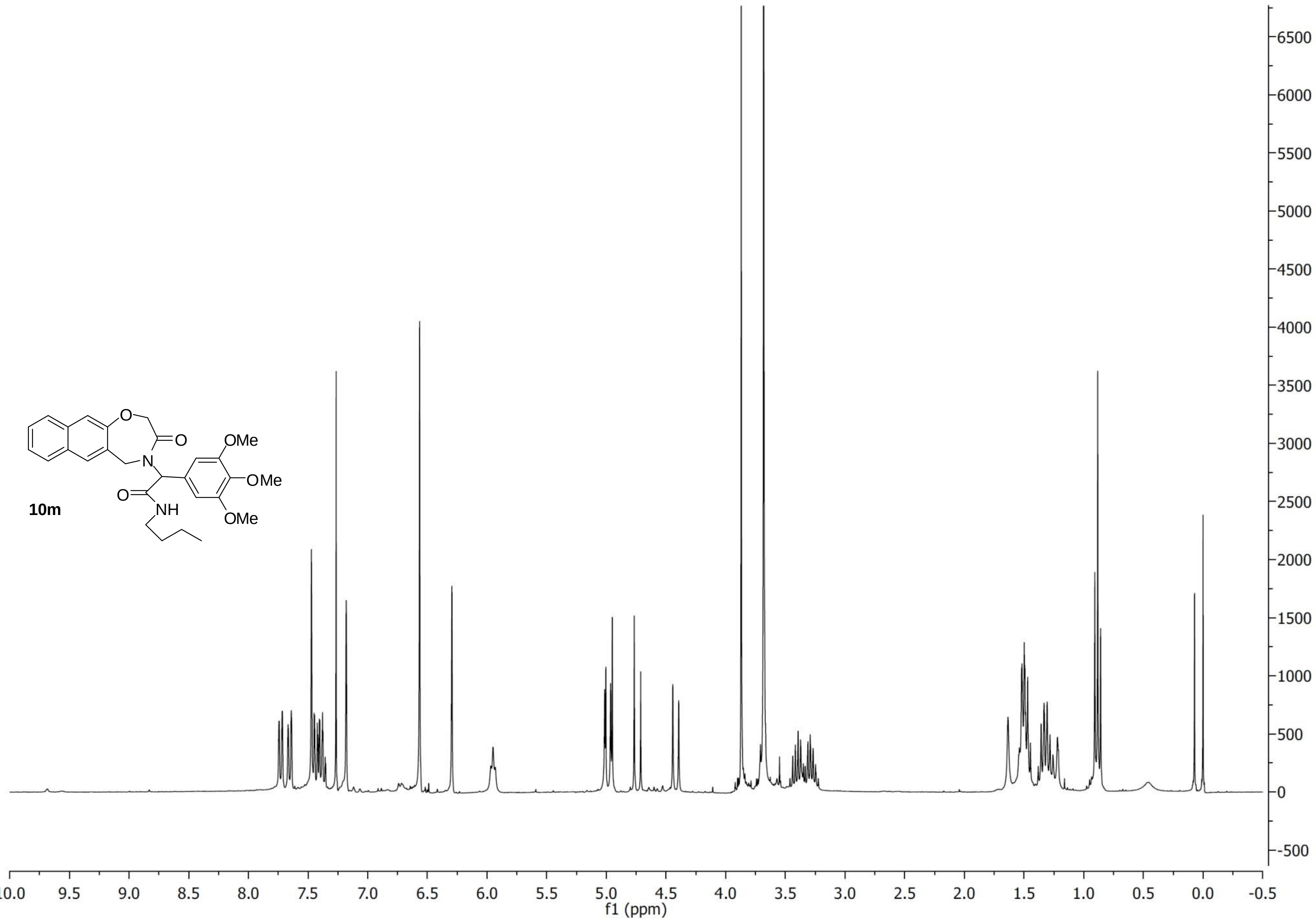
OMe
OMe
OMe
O
O
N
O
NH
10m
6500
6000
5500
5000
4500
4000
3500
3000
2500
2000
1500
1000
500
0
-500
10.0 9.5 9.0 8.5 8.0 7.5 7.0 6.5 6.0 5.5 5.0 4.5 4.0 3.5 3.0 2.5 2.0 1.5 1.0 0.5 0.0 -0.5
f1 (ppm)

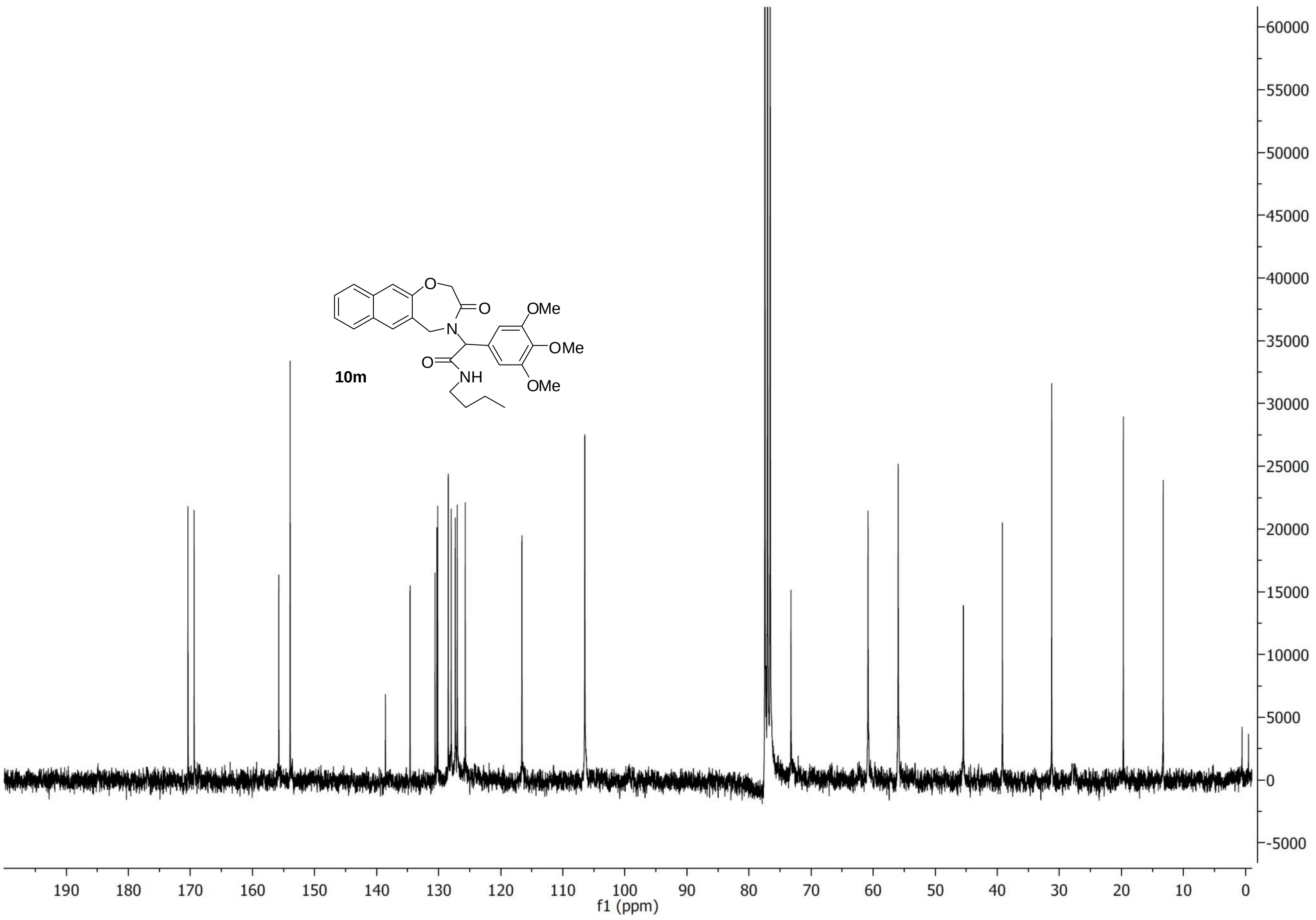
OMe
OMe
OMe
O
O
N
O
NH
10m
60000
55000
50000
45000
40000
35000
30000
25000
20000
15000
10000
5000
0
-5000
190
180
170
160
150
140
130
120
110
100
90
80
70
60
50
40
30
20
10
0
f1 (ppm)

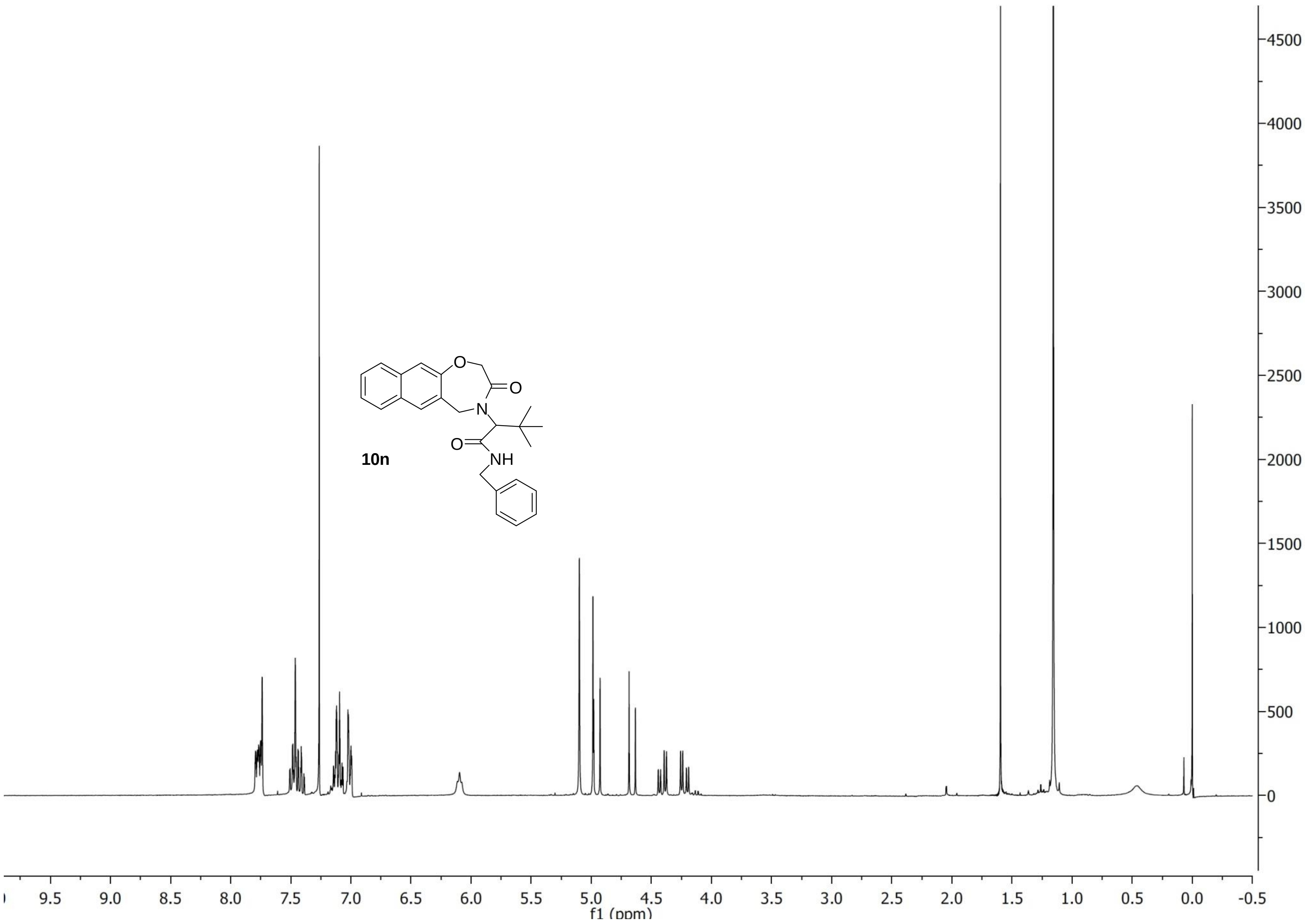
O
O
N
O
NH
10n
4500
4000
3500
3000
2500
2000
1500
1000
500
0
9.5 9.0 8.5 8.0 7.5 7.0 6.5 6.0 5.5 5.0 4.5 4.0 3.5 3.0 2.5 2.0 1.5 1.0 0.5 0.0 -0.5
f1 (ppm)

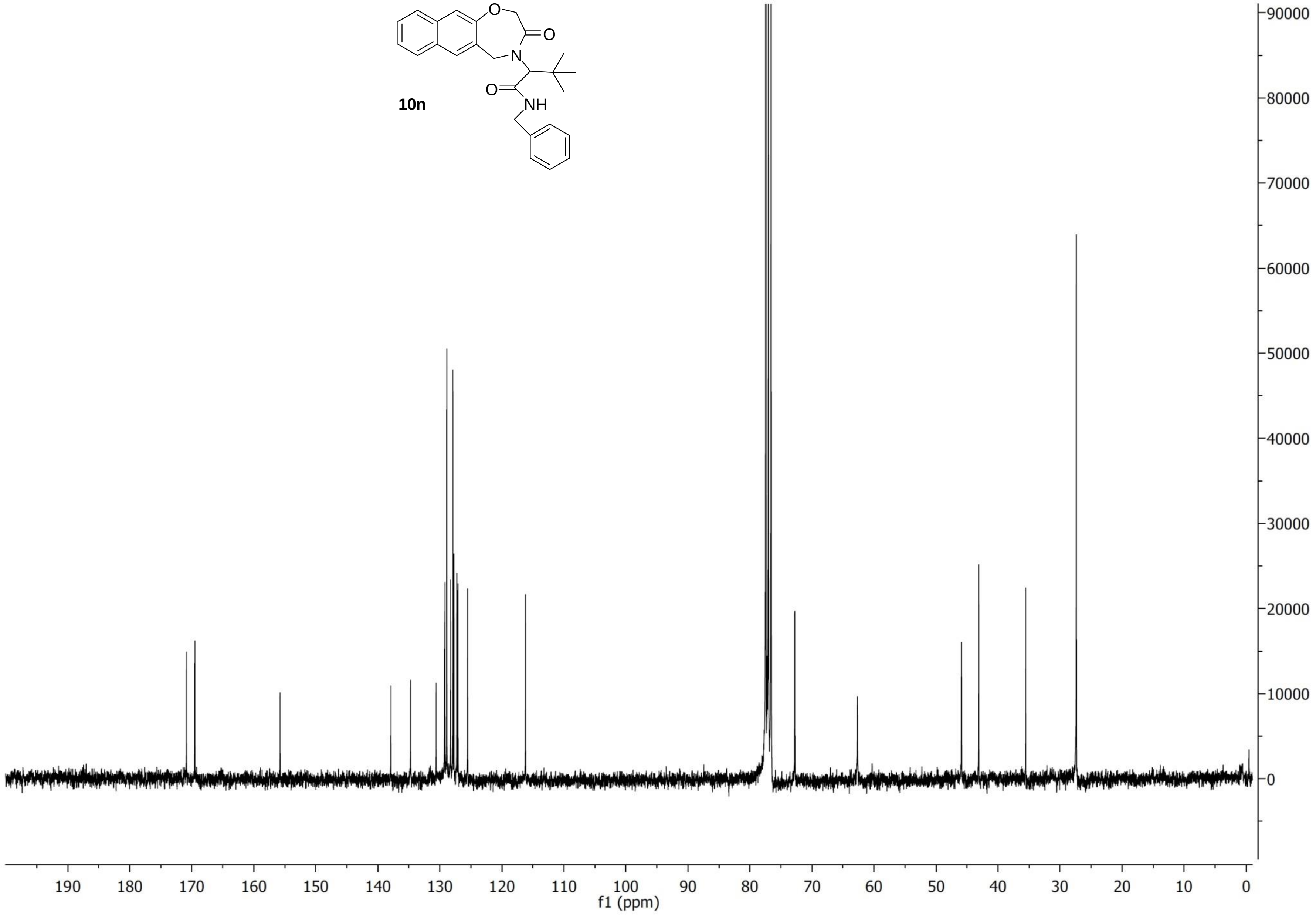
10n
O
O
N
O
NH
90000
80000
70000
60000
50000
40000
30000
20000
10000
0
190 180 170 160 150 140 130 120 110 100 90 80 70 60 50 40 30 20 10 0
f1 (ppm)
